# Quantitative site- and structure-specific N-glycoproteomics characterization of differential N-glycosylation in MCF-7/ADR cancer stem cells

**DOI:** 10.1186/s12014-020-9268-7

**Published:** 2020-02-05

**Authors:** Feifei Xu, Yue Wang, Kaijie Xiao, Yechen Hu, Zhixin Tian, Yun Chen

**Affiliations:** 1 School of Pharmacy, Nanjing Medical University, Nanjing, 211166 China; 2 School of Chemical Science & Engineering, Shanghai Key Laboratory of Chemical Assessment and Sustainability, Tongji University, Shanghai, 200092 China

**Keywords:** Cancer stem cells, Quantitative site- and structure-specific N-glycoproteomics, Intact N-glycopeptides, GPSeeker

## Abstract

**Background:**

Cancer stem cells (CSCs) are reported to be responsible for tumor initiation, progression, metastasis, and therapy resistance where P-glycoprotein (P-gp) as well as other glycoproteins are involved. Identification of these glycoprotein markers is critical for understanding the resistance mechanism and developing therapeutics.

**Methods:**

In this study, we report our comparative and quantitative site- and structure-specific N-glycoproteomics study of MCF-7/ADR cancer stem cells (CSCs) vs. MCF-7/ADR cells. With zic-HILIC enrichment, isotopic diethyl labeling, RPLC–MS/MS (HCD) analysis and GPSeeker DB search, differentially expressed N-glycosylation was quantitatively characterized at the intact N-glycopeptide level.

**Results:**

4016 intact N-glycopeptides were identified with spectrum-level FDR ≤ 1%. With the criteria of ≥ 1.5 fold change and p value < 0.05, 247 intact N-glycopeptides were found differentially expressed in MCF-7/ADR CSCs as putative markers. Raw data are available via ProteomeXchange with identifier PXD013836.

**Conclusions:**

Quantitative site- and structure-specific N-glycoproteomics characterization may help illustrate the cell stemness property.

## Introduction

Aberrant N-glycosylation is increasingly recognized as one of the most important biochemical changes involved in tumorigenesis and metastasis [1–4]; most of the FDA approved cancer biomarkers are glycoproteins. Cancer stem cells (CSCs) are a small population of stem-like cells and reported to be responsible for tumor initiation, progression, metastasis, and therapy resistance where P-glycoprotein (P-gp) as well as other glycoproteins are involved [5–9]. Identification of these glycoprotein markers is critical for understanding the resistance mechanism and developing therapeutics.

In 2006, Fujiwara et al. analyzed gene expression profiles of the ATP-binding cassette (ABC) transporters in breast cancer patients who underwent sequential weekly paclitaxel/FEC neoadjuvant chemotherapy using oligonucleotide microarrays; and six ABC transporters (ABCC5, ABCA12, ABCA1 ABCC13, ABCB6 and ABCC11) were found to be significantly up-regulated in the residual disease (p < 0.05) [10].

In 2015, Li et al. found that isocyclopamine reversed doxorubicin resistance of MCF-7/ADR cells via down-regulation of the cancer stem-like cells and modulation on both ABCB1 and ABCG2 transporters [11]. In 2016, Han et al. found that knockdown of SALL4 reversed the resistance of MCF-7/ADR cells to doxorubicin together with down-regulation of ABCG2 and c-myc [12]. In 2017, Xing et al. found that ALDH1 and ABCG2 were enhanced in primary foci and metastatic lymph node from patients with triple-negative breast cancer with qRT-PCR, western blotting and MTT assay of mRNA expression, protein expression and proliferation of MDA-MB-231 cells, respectively; and the authors proposed that ALDH1 and ABCG2 may affect the drug resistance [13]. In 2018, Bogusha et al. found that induction of the epithelial-mesenchymal transition process made bigger contribution to the drug resistance of MCF-7/ADR cells than the ABC transporter’s overexpression, because differential expression of vimentin is much higher than that of P-gp as measured by immunofluorescent staining with antibodies [14].

Here, to explore the N-glycosylation CSCs markers and discover the drug-resistant mechanism, we report our comparative N-glycoproteomics study of MCF‐7/ADR cancer stem cells (CSCs) vs. MCF-7/ADR cells; the culture, sorting, and detection of the cells were well-defined and controlled, thus the two types of cells were pure and good models for exploring the role of CSCs in drug resistance. With zic-HILIC enrichment, isotopic diethyl labeling, RPLC-MS/MS (HCD) analysis and GPSeeker DB search, differentially expressed N-glycosylation was quantitatively characterized at the intact N-glycopeptide level.

## Experimental

### Chemicals and reagents

Dithiothreitol (DTT, 3483-12-3), iodoacetamide (IAA, 144-48-9), 2,2,2-trifluoroethanol (TFE,  ≥ 99%, 75-89-8), sodium cyanoborohydride (25895-60-7), acetaldehyde- ^13^C_2_ (99 atom  % ^13^C, 1632-98-0), ammonium hydroxide solution (28–30% NH_3_ basis, 1336-21-6), trifluoroacetic acid (TFA, 99%, 76-05-1), formic acid (FA, 64-18-6), trypsin and all HPLC solvents were purchased from Sigma-Aldrich (St. Louis, MO, USA). Acetaldehyde solution (40% in H_2_O, 75-07-0) was obtained from General Reagent (Shanghai). Ultrapure water was produced on site by Millipore Simplicity System (Billerica, MA, USA).

### Cell culture of MCF-7/ADR and MCF-7/ADR CSCs

Drug-resistant cell line MCF-7/ADR was cultured using DMEM (Thermo Scientific Hyclone, MA, USA) supplied with 10% fetal bovine serum and 100 U/mL penicillin and 100 μg/mL streptomycin at 37 °C and 5% CO_2_. To maintain a highly drug-resistant cell population, MCF-7/ADR cells were periodically reselected by growing them in the presence of 1000 ng/mL Adriamycin. Experiments were performed using the cells incubated without DOX for 48 h. CD24- and CD44-microbeads antibodies (Miltenyi Biotec, Germany) were used for cell sorting of Breast Cancer Stem Cells (BCSCs) [15]. Briefly, 10^7^ total MCF-7/ADR cells were incubated with the above antibodies on ice for 40 min. After washing with cold PBS, CD44 + CD24 −/low BCSCs named MCF-7/ADR CSCs were purified from MCF-7/ADR cell lines. The characteristics of MCF-7/ADR CSCs were regularly detected by flow cytometry and maintained into ultra-low attachment six well plates (Corning, New York, USA) in MammoCult™ Human Medium Kit (Stem cell technologies, Vancouver, Canada) according to manufacturer’s guideline [16].

### Protein extraction and trypsin digestion

Cells (either MCF-7/ADR or MCF-7/ADR CSCs, two 10 cm-dishes) were disrupted on ice in 1 mL of lysis buffer (0.1 M Tris/HCl, 4% SDS, pH 8.0) by sonication (Ningbo Scientz Biotechnology CO,.LTD, China) for 15 min. The whole cell lysates were centrifuged at 14,000 rpm and 4 °C for 15 min, and the supernatant protein mixtures were collected. After acetone precipitation, proteins were dissolved in 1 mL of 8 M urea and were diluted in 10 mL ultrapure water. Protein concentration was determined by BCA assay (SK3021, Sangon Biotech, Shanghai, China).

One mg of proteins were reduced with 20 mM DTT (20 min, 55 °C), alkylated with 20 mM iodoacetamide (in the dark, 30 min, RT), and digested with trypsin (1:50 w/w, 37 °C, 16 h, stopping reagent 0.5% TFA). The digests were desalted using house-made C18-tip and eluted with 400 μL of 50% acetonitrile (ACN) and 400 μL of 80% ACN. Desalted peptides were concentrated and stored at − 20 °C for further use.

### ZIC-HILIC enrichment of intact N-glycopeptides

Intact N-glycopeptides were enriched using ZIC-HILIC (zwitterionic type of hydrophilic interaction chromatography) particles [17]. Briefly, desalted peptides were redissolved in 80% ACN with 1% TFA and loaded onto a house-made pipette tip containing 30 mg ZIC-HILIC particles (Merk Millipore, 5 μm, 200 Å) which were pre-equilibrated with 0.1% TFA and 80% ACN with 1% TFA. After sample binding, the tip was washed using 800 μL 80% ACN with 1% TFA. Enriched N-glycopeptides were eluted with 300 μL 0.1% TFA and 100 μL 50 mM NH_4_HCO_3_, dried in a vacuum concentrator, and stored at − 20 °C for further use.

### Isotopic diethyl labelling of the enriched intact N-glycopeptides

Stock solution of NaBH_3_CN (600 mM), CH_3_CHO (20%, w/w), ^13^CH_3_^13^CHO (20%, w/w), NH_4_OH (10%, v/v) and formic acid (5%, v/v) were freshly made. Diethylation of N-terminal and lysine amino groups with CH_3_CHO and NaBH_3_CN was carried out using the same protocol as reported for peptides [18]. Two identical aliquots of MCF-7/ADR and MCF-7/ADR CSCs N-glycopeptides were enriched and re-suspended in 100 μL TFE, and 8 μL 20% acetaldehyde or acetaldehyde-^13^C_2_ was added. Subsequently, 8 μL freshly prepared 600 mM NaBH_3_CN was added and incubated at 37 °C for 1 h, and the reaction was quenched with incubation with 8 μL 4% (v/v) NH_4_OH for 1 min followed by addition of 6 μL 5% (v/v) FA. After concentrated, the labeled N-glycopeptides were desalted using house-made C18-tip and eluted with 250 μL of 50% ACN and 250 μL of 80% ACN. Desalted peptides were concentrated and re-suspended in ultrapure water for further analysis.

### C18-RPLC-MS/MS (HCD) analysis of the 1:1 mixture of the labelled intact N-glycopeptides of MCF-7/ADR and MCF-7/ADR CSCs

For one RPLC-MS/MS analysis, an equivalent of 200 μg proteins from MCF-7/ADR or MCF-7/ADR CSCs were used as starting material (before ZIC-HILIC enrichment). The N-glycopeptides were separated on a 70 cm long analytical column (360 μm o.d. × 75 μm i.d.) packed with C18 particles (300 Å, 5 μm) on a Dionex Ultimate 3000 RSLC nano-HPLC system (Thermo Fisher Scientific) without any trap column. Buffer A is mixture of 99.8% H_2_O and 0.2% FA; buffer B is mixture of 95.0% ACN, 4.8% H_2_O, and 0.2% FA. Elution at a constant flow of 300 nL/min was conducted at the following gradient. The gradient was 4 h in total for complex samples: 2% buffer B for 25 min for sample-loading and 2–40% B in 135 min, followed by an increase to 95% B in 5 min, held for another 5 min and held for 2% B for the last 65 min for equilibration.

Eluted N-glycopeptides were detected online with nano-ESI tandem mass spectrometry using a Q Exactive mass spectrometer (Thermo Fisher Scientific, San Jose, CA, USA). MS spectra were acquired in the 700–2000 *m/z* range using a mass resolution 70 k (*m/z* 200). For MS/MS spectra, the mass resolution was set at 17.5 k. Fragmentation was obtained in a data-dependent mode (Top20) with higher-energy collisional dissociation (HCD). The automatic gain control (AGC) target value and maximum injection time were placed at 2 × 10^5^ and 50 ms for MS and at 5 × 10^5^ and 250 ms for MS/MS scans. Isolation window and dynamic exclusion were set at 3.0 *m/z* and 20.0 s. Stepped normalized collision energies was optimally set at 20.0%, 30.0%, and 40.0%. The temperature of the ion transfer capillary was set to 280 °C. The spray voltage was set to 2.8 kV.

### Database search and identification of intact N-glycopeptides in MCF-7/ADR and MCF-7/ADR CSCs using intact N-glycopeptide search engine GPSeeker

The RPLC-MS/MS (HCD) datasets were searched by DB search engine GPSeeker for intact N-glycopeptide identification with FDR control; the details have been reported elsewhere and only a brief description is given here. Four theoretical customized human intact N-glycopeptides databases of two directions (forward and decoy) and two labels (light and heavy diethylation) were first created, and each dataset was searched against the four databases independently. The search parameters for the precursor and fragment ions are isotopic abundance cutoff (IPACO), isotopic peak *m/z* deviation (IPMD), and isotopic abundance deviation (IPAD); the adopted IPACO, IPMD, IPAD values for both the precursor and the fragment ions are 40%, 20 ppm, and 50%, respectively. Initial GPSMs were obtained with the following refinement criteria: Y1 ions, Top4; minimal percentage of matched fragment ions of N-glycosite-containing peptides,  ≥ 10%; minimal matched product ions of N-glycan,  ≥ 1; TopN hits, N = 2 (top1 hits have the lowest P score). For each dataset, the target and decoy GPSMs from search of the four databases were combined and ranked with increasing P score, and a cutoff P score was then chosen to achieve spectrum-level FDR ≤ 1%. Target GPSMs with P scores lower than the cutoff value were grouped with the criteria of “peptide sequence, N-glycosite, and N-glycan linkage” for removal of duplicates and generation of the final list of intact N-glycopeptide IDs.

### Relative quantitation of differentially expressed intact N-glycopeptides in MCF-7/ADR CSCs relative to MCF-7/ADR using the quantitation module GPSeekerQuan in GPSeeker

Relative quantitation of the identified intact N-glycopeptides was carried out using GPSeekerQuan. A mass tolerance of 20 ppm and mass difference of 4.01344 Da were adopted for the search of the paired isotopic envelopes of the precursor ions in the MS spectra; in each isotopic envelope, top3 isotopic peaks were adopted. For each intact N-glycopeptide ID, all the six isotopic peaks are required to be observed for each pair of isotopic envelope; the peak abundance of the three isotopic peaks in each isotopic envelop was summed to obtain the relative ratio (MCF-7/ADR CSCs to MCF-7/ADR). At least two ratios need to be observed among the three technical replicates. For the intact N-glycopeptides quantitated at least twice, the *p* value was calculated using t-test [19]; and the intact N-glycopeptides with a fold change of no less than 1.5 and p value no bigger than 0.05 were classified as differentially expressed intact N-glycopeptides.

## Results

### Qualitative IDs

**Fig. 1:**
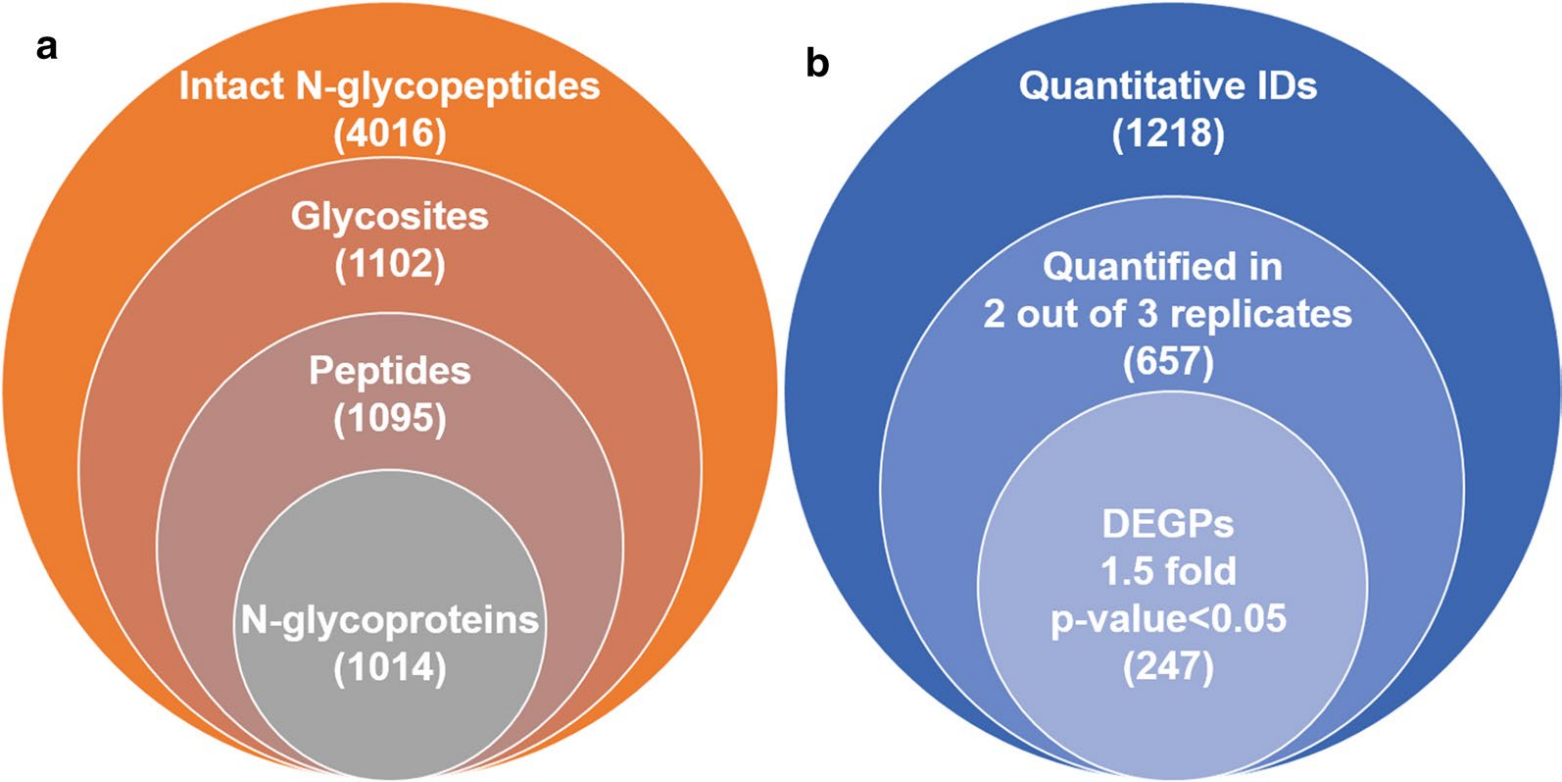
Intact N-glycopeptides identified (FDR ≤ 1%; **a**) and differentially expressed N-glycopeptides quantified (two out of three technical replicates, ≥ 1.5 fold, p < 0.05; **b**) from C18-RPLC-MS/MS (HCD) analysis of the 1:1 mixture of isotopically diethylated intact N-glycopeptides from MCF7/ADR and MCF7/ADR CSCs

With ZIC-HILIC enrichment, isotopically diethyl labeling, intact N-glycopeptides from MCF-7/ADR cells and MCF-7/ADR CSCs were mixed in 1:1 ratio and then online analyzed using C18-RPLC-nanoESI-MS/MS (HCD) to obtain three technical replicates (TR1, TR2 and TR3). The base-peak chromatograms from the three technical replicates are shown in Additional file 1: Figure S1. With target and decoy database searches using intact N-glycopeptide search engine GPSeeker, spectrum-level FDR control (≤ 1%) and duplicates removal, identified in total from the three technical replicates were 4016 intact N-glycopeptides corresponding to 1102 N-glycosites, 1095 unique peptides and 1014 intact N-glycoproteins (Fig. 1a), and 86 putative N-glycan linkages from 36 monosaccharide compositions. Among the 4016 intact N-glycopeptide IDs, 1847 were identified with glycoform score ≥ 1, i.e., more than one structure-diagnostic ions were identified for the N-glycan linkage structure in the matched fragment ions for each ID. Statistical analysis of the 1847 intact N-glycopeptides IDs shows the microheterogeneity of more than one glycoforms per N-glycosite is common (Additional file 1: Figure S2). For each of these 4016 intact N-glycopeptides, the detailed tabular information of dataset number, spectrum index, retention time, precursor ion (experimental and theoretical *m/z*, z, IPMD), accession number, peptide sequence, glycosite, monosaccharide composition, glycan primary structure in the format of one-line text, − log(P score), glyco-bracket, and GF score is listed in Additional file 2: Table S1.

### Quantitative results and differentially expressed intact N-glycopeptides

**Fig. 2:**
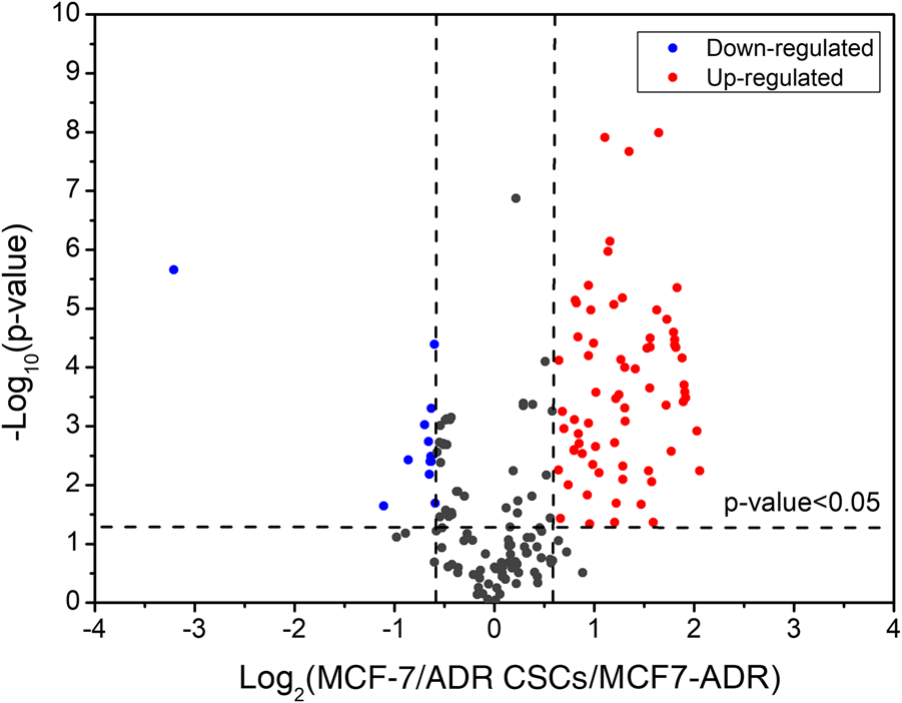
Volcano plot of the quantified (two out of three technical replicates) intact N-glycopeptides from C18-RPLC-MS/MS (HCD) analysis of the 1:1 mixture of isotopically diethylated intact N-glycopeptides from MCF7/ADR and MCF7/ADR CSCs

**Fig. 3:**
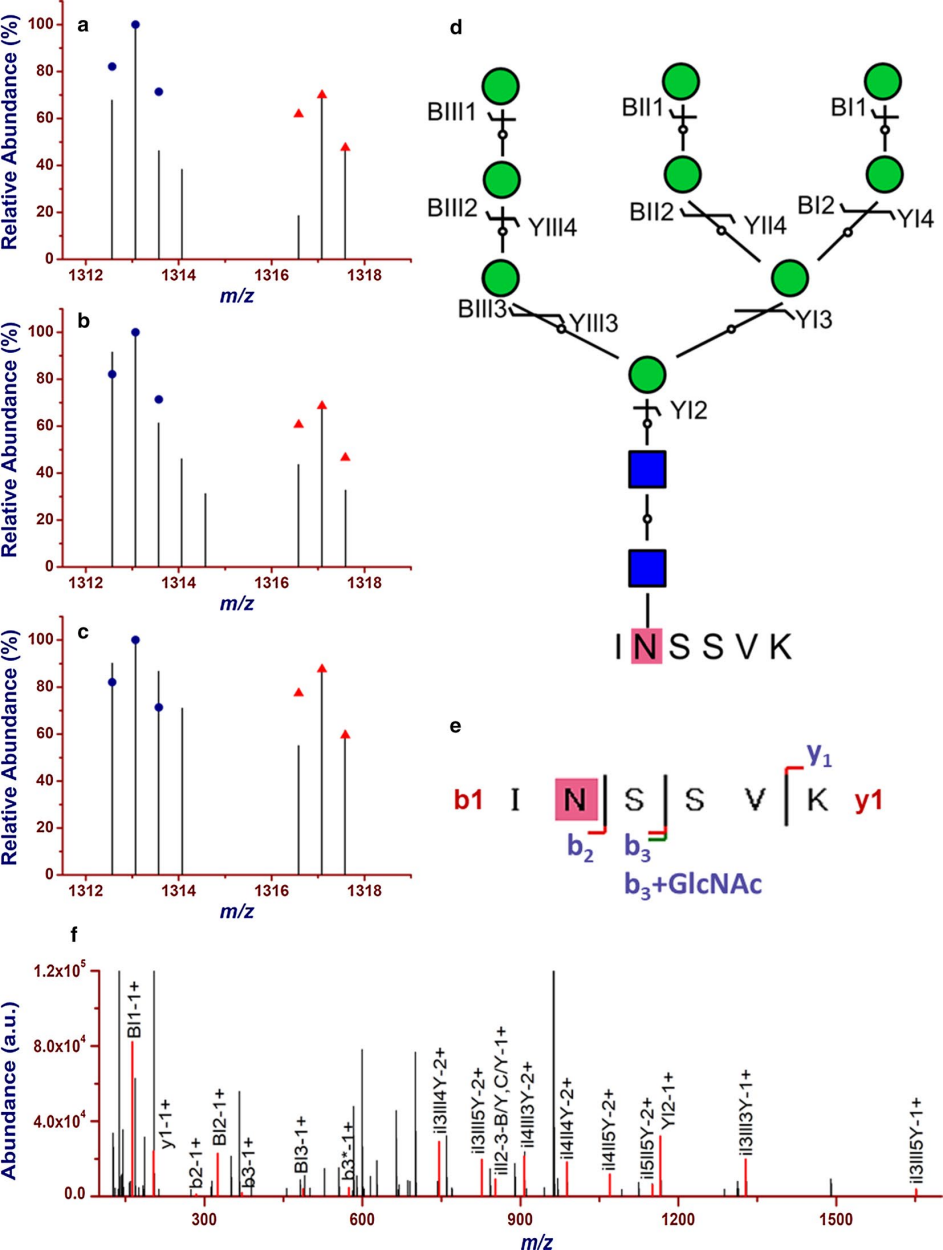
Quantification of down-regulation (0.56 ± 0.07) of intact N-glycopeptide INSSVK_N2H9F0S0 from N-glycoprotein RalBP1-associated Eps domain-containing protein 1 (Q96D71, N-glycosite N498) in MCF-7/ADR CSCs relative to MCF-7/ADR cells. (**a**–**c**) the isotopic envelope fingerprinting maps of the precursor ions in the three technical replicates; **d** selective fragmentation and the graphical fragmentation map of N-glycan moiety with the peptide backbone, **e** fragmentation and the graphical fragmentation map of the peptide backbone with one core GlucNAc, and **f** the annotated MS/MS spectrum with the matched fragment ions in representative spectrum 19,803 of TR1

**Fig. 4:**
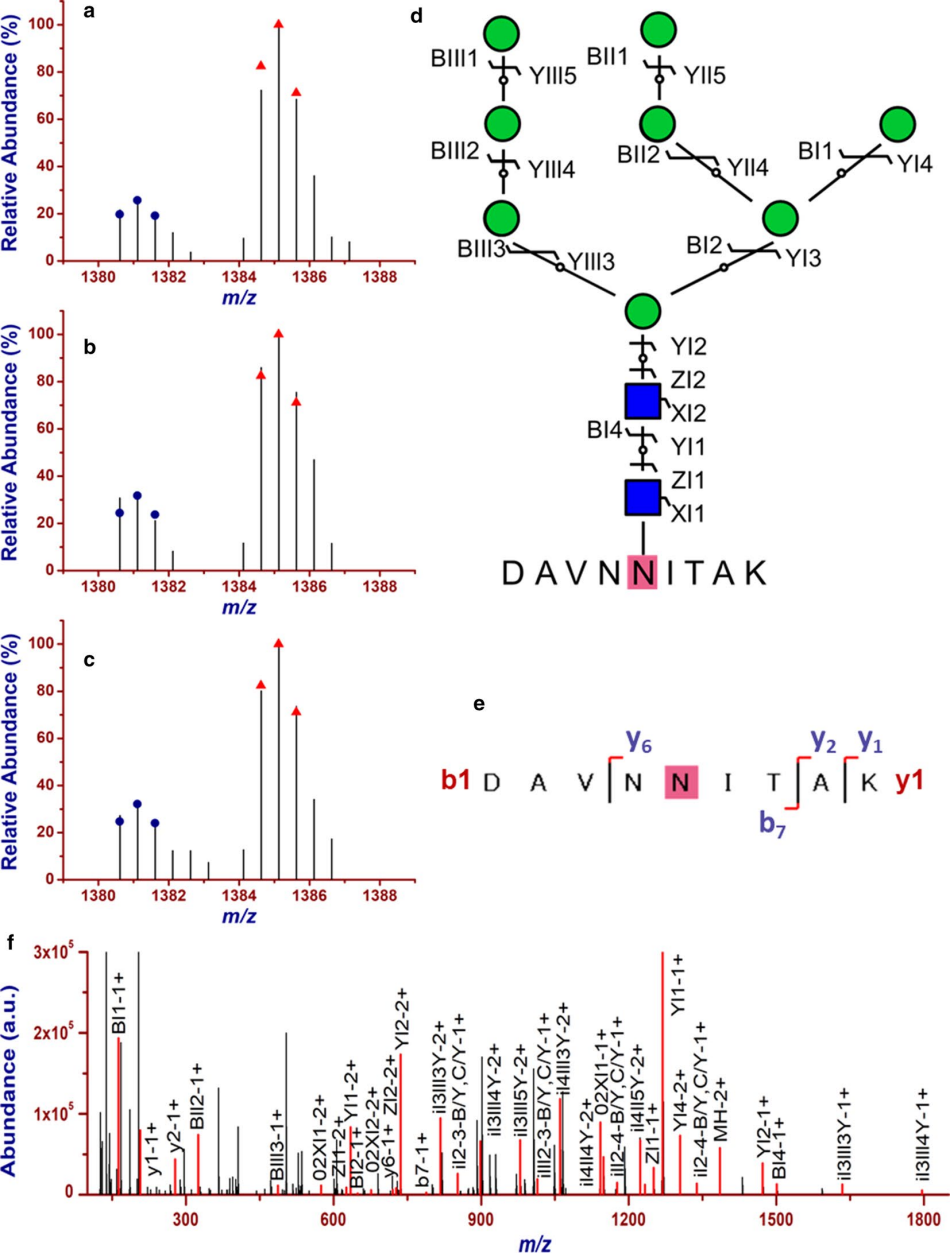
Quantification of up-regulation of (3.54 ± 0.33) of intact N-glycopeptide DAVNNITAK_N2H8F0S0 from N-glycoprotein Voltage-dependent calcium channel subunit alpha-2/delta-1 (P54289, N-glycosite N324) in MCF-7/ADR CSCs relative to MCF-7/ADR cells. (**a**– **c**) the isotopic envelope fingerprinting maps of the precursor ions in the three technical replicates; **d** selective fragmentation and the graphical fragmentation map of N-glycan moiety with the peptide backbone, **e** fragmentation and the graphical fragmentation map of the peptide backbone with one core GlucNAc, and **f** the annotated MS/MS spectrum with the matched fragment ions in representative spectrum 17,216 of TR1

The abundance of the 4016 intact N-glycopeptide IDs together with their isotopic pairs (6xn Da) in the corresponding MS spectra were then searched with GPSeekerQuan. With the criteria of observation of all the six most abundant isotopic peaks, 1218 IDs were quantified at least once and 657 at least twice out of the three technical replicates (Additional file 3: Table S2). Further with the criteria of  ≥ 1.5 fold change and p < 0.05, 247 intact N-glycopeptides were found differentially expressed (Fig. 1b) with an average RSD of 7.60%, where 51 were down-regulated and 196 up-regulated (Fig. 2). For example, intact N-glycopeptide INSSVK_N2H9F0S0 from N-glycosite N498 of N-glycoprotein RalBP1-associated Eps domain-containing protein 1 (REPS1_HUMAN, Q96D71) was found to be down regulated (0.56 ± 0.07) in MCF-7/ADR CSCs relative to MCF-7/ADR cells (Fig. 3); intact N-glycopeptide DAVNNITAK_N2H8F0S0 from N-glycosite N324 of N-glycoprotein Voltage-dependent calcium channel subunit alpha-2/delta-1 (CA2D1_HUMAN, P54289) was found to be up regulated (3.54 ± 0.33) in MCF-7/ADR CSCs relative to MCF-7/ADR cells (Fig. 4). Most of the differentially expressed intact N-glycopeptides (DEGPs) we previously quantified in MCF-7 CSCs (relative to MCF-7 cells) were found to have no significant differential expression in MCF-7/ADR CSCs (relative to MCF-7/ADR cells) in this study except for the intact N-glycopeptide SLSNSTAR_N2H5F0S0 (Serpin H1, P50454, N120) (Additional file 1: Figure S3).

**Fig. 5:**
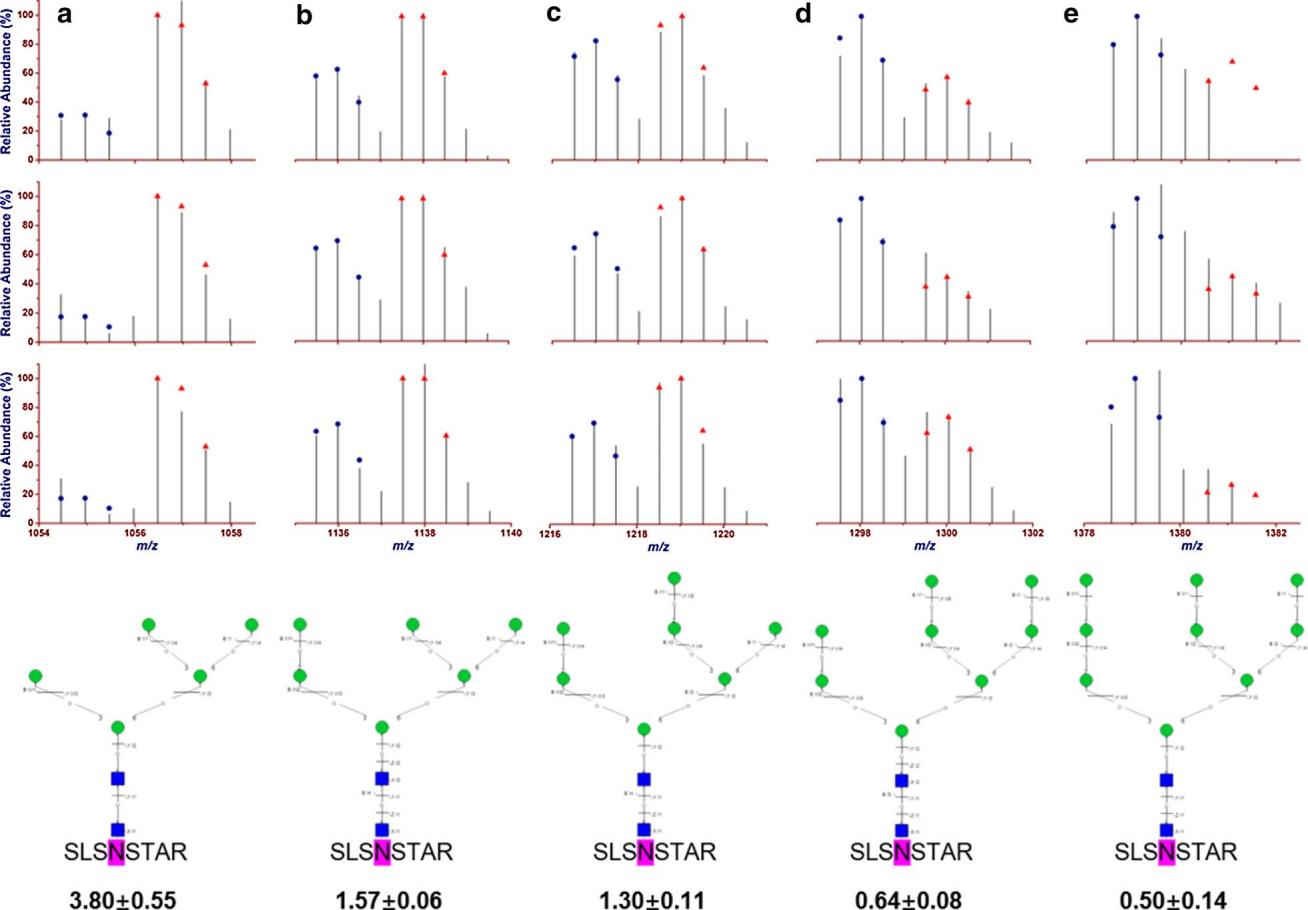
Continuous change of differential expression vs. the high-mannose N-glycan size for intact N-glycopeptide series SLSNSTAR_N2HxF0S0 (x = 5, 6, 7, 8, 9; from left to right: **a**–**e**) with three technical replicates (from up to down) identified from N-glycosite N120 of intact N-glycoprotein Serpin H1 (P50454); SLSNSTAR_N2H9F0S0 were quantified with the Top2 isotopic peaks of the last two replicates

For intact N-glycoprotein serpin H1 (P50454), a series of five high-mannose intact N-glycopeptides SLSNSTAR_N2HxF0S0 (x = 5, 6, 7, 8, 9) were identified on N-glycosite N120; a continuous transition from up-regulation at x = 5 to down-regulation at x = 9 in MCF-7/ADR CSCs vs. MCF-7/ADR was observed (Fig. 5). Additional file 1: Figure S4 shows that the intact N-glycopeptide SLSNSTAR_N2H5F0S0 was found to be up-regulated in both cell lines (4.23 ± 0.71 in MCF-7 CSCs and 3.80 ± 0.55 in MCF-7/ADR CSCs).

### Molecular functions, cellular components and biological processes of DEGPs

**Fig. 6:**
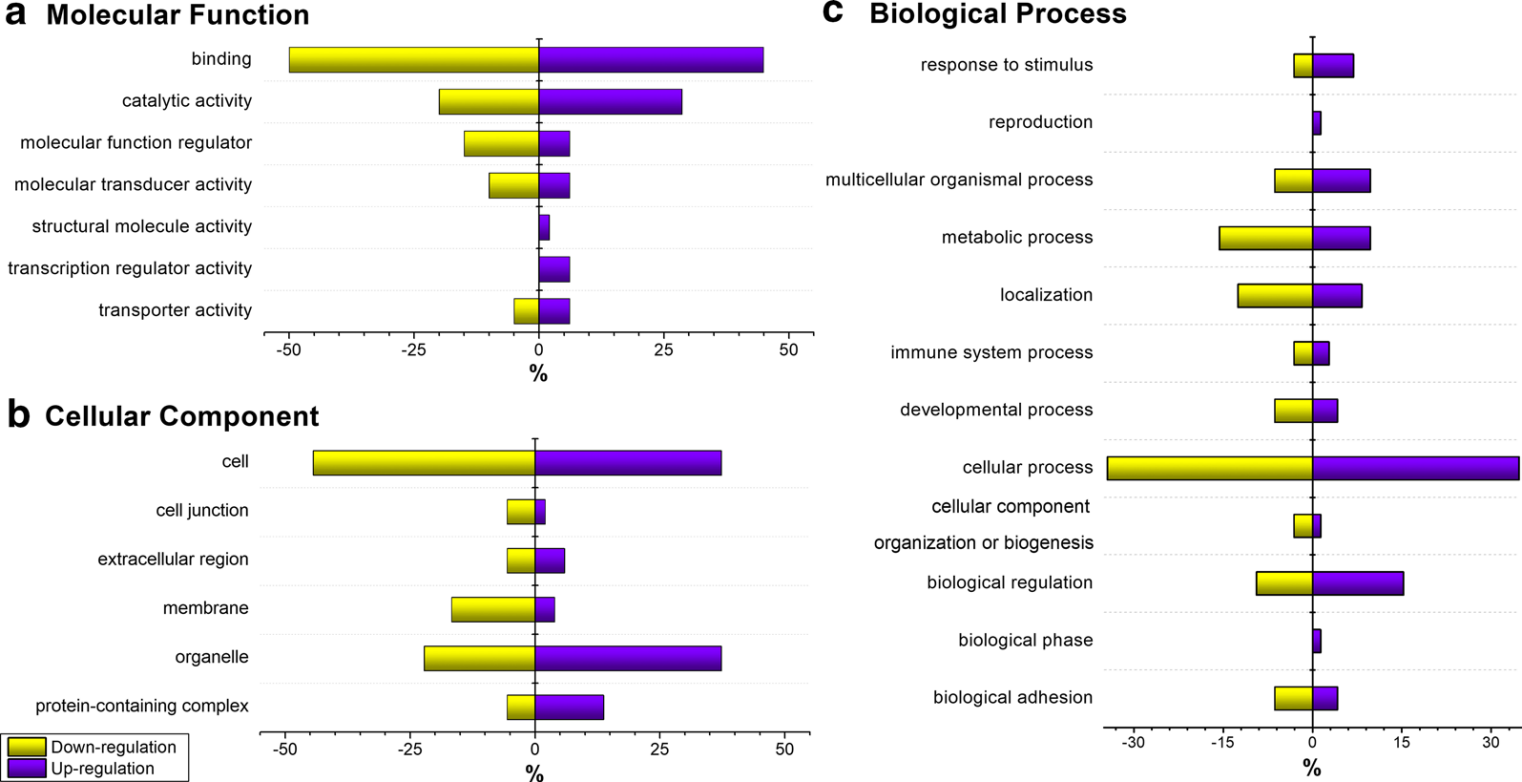
Gene Ontology analysis of the N-glycoproteins corresponding to the differentially expressed intact N-glycopeptides in MCF-7/ADR CSCs relative to MCF-7/ADR. **a** Molecular function; **b** cellular component; **c** biological process

Gene ontology (GO) analysis using PANTHER (protein annotation through evolutionary relationship) classification system (http://pantherdb.org/) was performed on the 247 differentially expressed intact N-glycopeptides in MCF-7/ADR CSCs and showed that in generally both up- and down-regulated N-glycopeptides participated in the cellular process, metabolic process and biological regulation; while their molecular functions were mainly binding, catalytic activity and molecular function regulator (Fig. 6). However, there were differences in the degree of enrichment between the two cell lines in processes and functions. For example, the up-regulated proteins were more active in the biological regulation, response to stimulus, multicellular organismal process and played more roles of catalytic activity, transporter activity and transcription regulator activity; while the down-regulated proteins performed more on binding, molecular transducer activity and molecular function regulator. These differences could be further explained by cellular components analysis. The up-regulated proteins were more likely to be found in organelle and protein-containing complex, while the down-regulated proteins were more concentrated on the cell membrane, which implied that more N-glycoproteins from MCF-7/ADR cells were used for cellular recognition and conjunction, and more N-glycosylation happened in the intracellular regions of MCF-7/ADR CSCs was to exercise their biological regulatory functions and response to stimulus.

## Discussion

### Drug-resistance N-glycosylation markers

Drug resistance is a major problem in cancer chemotherapy. Aberrant glycosylation has been known to be associated with cancer chemoresistance. Therefore, identifying those glycoproteins that are expressed specifically by tumor cells and correlate with chemoresistance is important.

Adenosine triphosphate-binding cassette (ABC) drug transporters function as drug efflux pumps which leads to drug resistance, and stem cells appear to express multiple ABC transport proteins. In this study, nine ABC proteins were either identified (ABCC5, ABCA4, ABCB9) or observed as GPSMs (ABCA12, ABCA13, ABCA2, ABCA3, ABCB1, ABCC9).

For ABCC5 (O15440), intact N-glycopeptide GANLSGGQRQR-N2H8F0S0 at N-glycosite N684 was identified from spectrum 17,391 of TR1, with a fold of 0.46 down-regulation in MCF-7/ADR CSCs (Additional file 1: Figure S5). This observation was in agreement with that ABCC5 was expressed in almost every human cancer cell line [20].

For ABCA4 (P78363), intact N-glycopeptide IMNVSGGPITR-N2H8F0S0 at N-glycosite N1588 was identified in spectrum 17,575 of TR2, with a fold of 0.22 down-regulation in MCF-7/ADR CSCs (Additional file 1: Figure S6). ABCC4 was reported to be decreased in the MCF7/AdVp3000 cells using total RNAs isolated from the parental cell line MCF7 and its derivative drug resistant cell line MCF7/AdVp3000 [21].

For ABCB9 (Q9NP78), intact N-glycopeptide VDFENVTFTYR-N2H8F0S0 at N-glycosite N508 was identified from spectrum 20,416 of TR3; and up-regulation of 1.89-fold quantitated by the single left peak was observe (Additional file 1: Figure S7). Up-regulation of ABCB9 mRNA was also detected in chorioamnionitis (p < 0.05) [22].

In summary, the above discussion demonstrated that our results were reliable. Moreover, for effective treatment, the distinction between cancer stem cells and stem cells should be found. Our results also gave a precise comparison of the drug-resistance N-glycosylation markers of MCF-7/ADR CSCs and MCF-7/ADR cells, which could provide more experimental information for further clinical treatment.

### CSC N-glycosylation markers

Markers located on the cell surface are often used to identify and enrich CSCs, and the expression of these markers is statistically related to the likelihood of cancer recurrence and overall patient survival. Therefore, CSC markers have a high clinical significance. Most of the markers currently used to identify CSC populations are glycoproteins, thus, elucidating the different expressed glycoproteins gave us hints to discover new knowledge to study CSCs.

Some common CSC markers are either quantified or identified in this study. For zinc finger protein GLI1, intact N-glycopeptide AFSNASDRAK-N2H8F0S0 on N-glycosite N344 was quantified to be up regulated by a fold of 2.66 ± 0.03 in MCF-7/ADR CSCs relative to MCF-7/ADR cells (Additional file 1: Figure S8). HEDGEHOG-GLI1 signalling was found previously to regulate human Glioma CSC self-renewal and tumorigenicity [23]. For CD63 antigen, intact N-glycopeptide NNHTASILDR-N2H8F0S0 on N-glycosite N130 was quantified to be up regulated by a fold of 3.39 ± 0.26 in MCF-7/ADR CSCs relative to MCF-7/ADR cells (Additional file 1: Figure S9). Over expression of CD63 protein as measured by immunohistochemistry was previously observed in glioblastomas and its role in stemness was suggested [24]. For CD13, intact N-glycopeptide AEFNITLIHPK-N2H7F0S0 on N-glycosite N234 was quantified to be up regulated by a fold of 2.30 ± 0.53 in MCF-7/ADR CSCs relative to MCF-7/ADR cells (Additional file 1: Figure S10). CD13 was previously identified as marker of semiquiescent liver CSCs [25]. For CD49F, intact N-glycopeptide ANHSGAVVLLKR-N2H6F0S0 on N-glycosite N323 was identified from spectrum 19,068 of TR2; and down-regulation of 0.77-fold was observed in MCF-7/ADR CSCs relative to MCF-7/ADR cells (Additional file 1: Figure S11). Up-regulation of CD49F was previously observed in normal adjacent tissues of patients with triple negative breast cancer and up-regulation of RNA as measured by qPCR was observed in breast cancer tissues [26].

Some new CSC markers are quantified in this study as well. Intact N-glycopeptide LNGTAKGER_N2H8F0S0 from N-glycosite 159 of N-glycoprotein segment polarity protein dishevelled homolog DVL-3 was quantified to be up-regulated in MCF-7/ADR CSCs; DVL3 participates in canonical Wnt signaling pathway. Intact N-glycopeptides SQNRSK_N2H8F0S0 from N-glycosite 302 of N-glycoprotein bone morphogenetic protein 7 (BMP7) and NATLAEQAK_N2H8F0S0 from N-glycosite 869 of N-glycoprotein hypoxia up-regulated protein 1 (HYOU1) were quantified to be up-regulated in MCF-7/ADR CSCs; BMP7 and HYOU1 involve in execution phase of apoptosis. Intact N-glycopeptide MSARNR_N2H8F0S0 from N-glycosite 176 of N-glycoprotein high mobility group protein B4 (HMGB4) was quantified to be up-regulated in MCF-7/ADR CSCs, and HMGB4 indicates activation of ERK pathway.

The overall observations provide a comprehensive list of putative N-glycoprotein biomarkers of MCF-7/ADR CSCs (relative to MCF-7/ADR cells), which is of great value in further elucidation of the biochemical mysteries of CSCs and discovery of effective cancer chemotherapy.

## Supplementary information

**Additional file 1: Figure S1.** MS-only base-peak chromatograms from RPLC-MS/MS analysis of the 1:1 mixture of the light- and heavy-diethylated intact N-glycopeptides enriched from MCF-7/ADR cells and MCF-7/ADR cancer stem cells; (**a**, **b**, **c**), three technical replicates. **Figure S2.** Counts of intact N-glycopeptides IDs with different number of glycoforms. **Figure S3.** Comparison of DEGPs in MCF-7 CSCs and MCF-7/ADR CSCs. **Figure S4.** Error curve of differentially expressed ratio(heavy/light) of five high-mannose intact N-glycopeptide SLSNSTAR_N2HxF0S0 (x = 5, 6, 7, 8, 9) on N-glycosite N120 of intact N-glycoprotein serpin H1 (P50454) in both MCF-7 CSCs and MCF-7/ADR CSCs. **Figure S5.** Identification of intact N-glycopeptide GANLSGGQRQR-N2H8F0S0 from multidrug resistance-associated protein 5 (ABCC5, MRP5_HUMAN, O15440, N684) from spectrum 17,391 of TR1, down-regulation of 0.46-fold in MCF-7/ADR CSCs vs. MCF-7/ADR cells was observed with the left two isotopic peaks. **Figure S6.** Identification of intact N-glycopeptide IMNVSGGPITR-N2H8F0S0 from retinal-specific ATP-binding cassette transporter (ABCA4_HUMAN, P78363, N1588) from spectrum 17,575 of TR2, down-regulation of 0.22-fold in MCF-7/ADR CSCs vs. MCF-7/ADR cells was observed with the left two isotopic peaks. **Figure S7.** Identification of intact N-glycopeptide VDFENVTFTYR-N2H8F0S0 from ATP-binding cassette sub-family B member 9 (ABCB9_HUMAN, Q9NP78, N508) from spectrum 20,416 of TR3, up-regulation of 1.89-fold was observed in MCF-7/ADR CSCs vs. MCF-7/ADR cells as quantitated with the left isotopic peak. **Figure S8.** Quantification of up-regulation (2.66 ± 0.03) of intact N-glycopeptide AFSNASDRAK-N2H8F0S0 (Zinc finger protein GLI1, P08151, N-glycosite N344) in MCF-7/ADR CSCs relative to MCF-7/ADR. **Figure S9.** Quantification of up-regulation (3.39 ± 0.26) of intact N-glycopeptide NNHTASILDR-N2H8F0S0 (CD63 antigen, P08962, N-glycosite N130) in MCF-7/ADR CSCs relative to MCF-7/ADR. **Figure S10.** Quantification of up-regulation (2.30 ± 0.53) of intact N-glycopeptide AEFNITLIHPK-N2H7F0S0 (CD13, P15144, N-glycosite N234) in MCF-7/ADR CSCs relative to MCF-7/ADR. **Figure S11.** Identification of intact N-glycopeptide ANHSGAVVLLKR-N2H6F0S0 from Integrin alpha-6 (CD49F, P23229, N323) from spectrum 19,068 of TR2, down-regulation of 0.77-fold was observed in MCF-7/ADR CSCs vs. MCF-7/ADR cells. **Figure S12**. Box plot of fold changes in glycopeptides from MCF-7/ADR CSCs relative to MCF-7/ADR.

**Additional file 2: Table S1**. The detailed tabular information of dataset number, spectrum index, retention time, precursor ion (experimental and theoretical *m/z*, z, IPMD), accession number, peptide sequence, glycosite, monosaccharide composition, glycan primary structure in the format of one-line text, − log(P score), glyco-bracket, and GF score for the 4016 intact N-glycopeptides identified from RPLC-MS/MS (HCD) analysis of the 1:1 mixture of isotopically diethylated intact N-glycopeptides enriched from MCF-7/ADR cancer stem cells and MCF-7/ADR cells. (Provided in a separate Excel file because of extra-ordinary length).

**Additional file 3: Table S2.** Differentially expressed intact N-glycopeptides (657) in MCF-7/ADR cancer stem cells (relative to MCF-7/ADR cells) quantitated at least twice out of the three technical replicates with ≥ 1.5-fold change and p < 0.05 from RPLC-MS/MS (HCD) analysis of the 1:1 mixture of isotopically diethylated intact N-glycopeptides. (Provided in a separate Excel file because of extra-ordinary length).

## Data Availability

The three RPLC-MS/MS (HCD) technical replicate datasets (.raw) are freely available at ProteomeXchange Consortium via the PRIDE partner repository [27]: PXD013836.
