# Supplementary material for: Quantitative site- and structure-specific N-glycoproteomics characterization of differential N-glycosylation in MCF-7/ADR cancer stem cells
Source: Clin Proteomics. 2020 Feb 5;17:3. doi: 10.1186/s12014-020-9268-7 (PMC7001331; doi:10.1186/s12014-020-9268-7)
Supplement: Supplementary file 1 — Additional file 1: Figure S1. MS-only base-peak chromatograms from RPLC-MS/MS analysis of the 1:1 mixture of the light- and heavy-diethylated intact N-glycopeptides enriched from MCF-7/ADR cells and MCF-7/ADR cancer stem cells; (a, b, c), three technical replicates. Figure S2. Counts of intact N-glycopeptides IDs with different number of glycoforms. Figure S3. Comparison of DEGPs in MCF-7 CSCs and MCF-7/ADR CSCs. Figure S4. Error curve of differentially expressed ratio(heavy/light) of five high-mannose intact N-glycopeptide SLSNSTAR_N2HxF0S0 (x = 5, 6, 7, 8, 9) on N-glycosite N120 of intact N-glycoprotein serpin H1 (P50454) in both MCF-7 CSCs and MCF-7/ADR CSCs. Figure S5. Identification of intact N-glycopeptide GANLSGGQRQR-N2H8F0S0 from multidrug resistance-associated protein 5 (ABCC5, MRP5_HUMAN, O15440, N684) from spectrum 17,391 of TR1, down-regulation of 0.46-fold in MCF-7/ADR CSCs vs. MCF-7/ADR cells was observed with the left two isotopic peaks. Figure S6. Identification of intact N-glycopeptide IMNVSGGPITR-N2H8F0S0 from retinal-specific ATP-binding cassette transporter (ABCA4_HUMAN, P78363, N1588) from spectrum 17,575 of TR2, down-regulation of 0.22-fold in MCF-7/ADR CSCs vs. MCF-7/ADR cells was observed with the left two isotopic peaks. Figure S7. Identification of intact N-glycopeptide VDFENVTFTYR-N2H8F0S0 from ATP-binding cassette sub-family B member 9 (ABCB9_HUMAN, Q9NP78, N508) from spectrum 20,416 of TR3, up-regulation of 1.89-fold was observed in MCF-7/ADR CSCs vs. MCF-7/ADR cells as quantitated with the left isotopic peak. Figure S8. Quantification of up-regulation (2.66 ± 0.03) of intact N-glycopeptide AFSNASDRAK-N2H8F0S0 (Zinc finger protein GLI1, P08151, N-glycosite N344) in MCF-7/ADR CSCs relative to MCF-7/ADR. Figure S9. Quantification of up-regulation (3.39 ± 0.26) of intact N-glycopeptide NNHTASILDR-N2H8F0S0 (CD63 antigen, P08962, N-glycosite N130) in MCF-7/ADR CSCs relative to MCF-7/ADR. Figure S10. Quantification of up-regulation (2.30 ± 0.5 [file 12014_2020_9268_MOESM1_ESM.doc]

Supporting Information

**Quantitative site- and structure-specific N-glycoproteomics characterization of differential N-glycosylation in MCF-7/ADR cancer stem cells**

Feifei Xu^1, #^, Yue Wang^2, #^, Kaijie Xiao^2^, Yechen Hu^1^, Zhixin Tian^2,*^, Yun Chen^1,*^

^1^School of Pharmacy, Nanjing Medical University, Nanjing 211166, China

^2^School of Chemical Science & Engineering, Shanghai Key Laboratory of Chemical Assessment and Sustainability, Tongji University, Shanghai 200092, China

# These authors contributed equally to this work.

*Correspondence to: Zhixin Tian, [zhixintian@tongji.edu.cn](mailto:zhixintian@tongji.edu.cn); Yun Chen, ychen@njmu.edu.cn

**Contents**

**Table S1.** The detailed tabular information of dataset number, spectrum index, retention time, precursor ion (experimental and theoretical *m/z*, z, IPMD), accession number, peptide sequence, glycosite, monosaccharide composition, glycan primary structure in the format of one-line text, −log(P score), glyco-bracket, and GF score for the 4,016 intact N-glycopeptides identified from RPLC-MS/MS (HCD) analysis of the 1:1 mixture of isotopically diethylated intact N-glycopeptides enriched from MCF-7/ADR cancer stem cells and MCF-7/ADR cells. (Provided in a separate Excel file because of extra-ordinary length.)

**Table S2.** Differentially expressed intact N-glycopeptides (657) in MCF-7/ADR cancer stem cells (relative to MCF-7/ADR cells) quantitated at least twice out of the three technical replicates with ≥1.5-fold change and p<0.05 from RPLC-MS/MS (HCD) analysis of the 1:1 mixture of isotopically diethylated intact N-glycopeptides. (Provided in a separate Excel file because of extra-ordinary length.)

**Figure S1.** MS-only base-peak chromatograms from RPLC-MS/MS analysis of the 1:1 mixture of the light- and heavy-diethylated intact N-glycopeptides enriched from MCF-7/ADR cells and MCF-7/ADR cancer stem cells; (A, B, C), three technical replicates.

**Figure S2.** Counts of intact N-glycopeptides IDs with different number of glycoforms.

**Figure S3.** Comparison of DEGPs in MCF-7 CSCs and MCF-7/ADR CSCs.

**Figure S4.** Error curve of differentially expressed ratio(heavy/light) of five high-mannose intact N-glycopeptide SLSNSTAR_N2HxF0S0 (x=5, 6, 7, 8, 9) on N-glycosite N120 of intact N-glycoprotein serpin H1 (P50454) in both MCF-7 CSCs and MCF-7/ADR CSCs.

**Figure S5.** Identification of intact N-glycopeptide GANLSGGQRQR-N2H8F0S0 from multidrug resistance-associated protein 5 (ABCC5, MRP5_HUMAN, O15440, N684) from spectrum 17391 of TR1, down-regulation of 0.46-fold in MCF-7/ADR CSCs vs. MCF-7/ADR cells was observed with the left two isotopic peaks.

**Figure S6.** Identification of intact N-glycopeptide IMNVSGGPITR-N2H8F0S0 from retinal-specific ATP-binding cassette transporter (ABCA4_HUMAN, P78363, N1588) from spectrum 17575 of TR2, down-regulation of 0.22-fold in MCF-7/ADR CSCs vs. MCF-7/ADR cells was observed with the left two isotopic peaks.

**Figure S7.** Identification of intact N-glycopeptide VDFENVTFTYR-N2H8F0S0 from ATP-binding cassette sub-family B member 9 (ABCB9_HUMAN, Q9NP78, N508) from spectrum 20416 of TR3, up-regulation of 1.89-fold was observed in MCF-7/ADR CSCs vs. MCF-7/ADR cells as quantitated with the left isotopic peak.

**Figure S8.** Quantification of up-regulation (2.66±0.03) of intact N-glycopeptide AFSNASDRAK-N2H8F0S0 (Zinc finger protein GLI1, P08151, N-glycosite N344) in MCF-7/ADR CSCs relative to MCF-7/ADR.

**Figure S9.** Quantification of up-regulation (3.39±0.26) of intact N-glycopeptide NNHTASILDR-N2H8F0S0 (CD63 antigen, P08962, N-glycosite N130) in MCF-7/ADR CSCs relative to MCF-7/ADR.

**Figure S10.** Quantification of up-regulation (2.30±0.53) of intact N-glycopeptide AEFNITLIHPK-N2H7F0S0 (CD13, P15144, N-glycosite N234) in MCF-7/ADR CSCs relative to MCF-7/ADR.

**Figure S11.** Identification of intact N-glycopeptide ANHSGAVVLLKR-N2H6F0S0 from Integrin alpha-6 (CD49F, P23229, N323) from spectrum 19068 of TR2, down-regulation of 0.77-fold was observed in MCF-7/ADR CSCs vs. MCF-7/ADR cells.

**Figure S12**. Box plot of fold changes in glycopeptides from MCF-7/ADR CSCs relative to MCF-7/ADR.


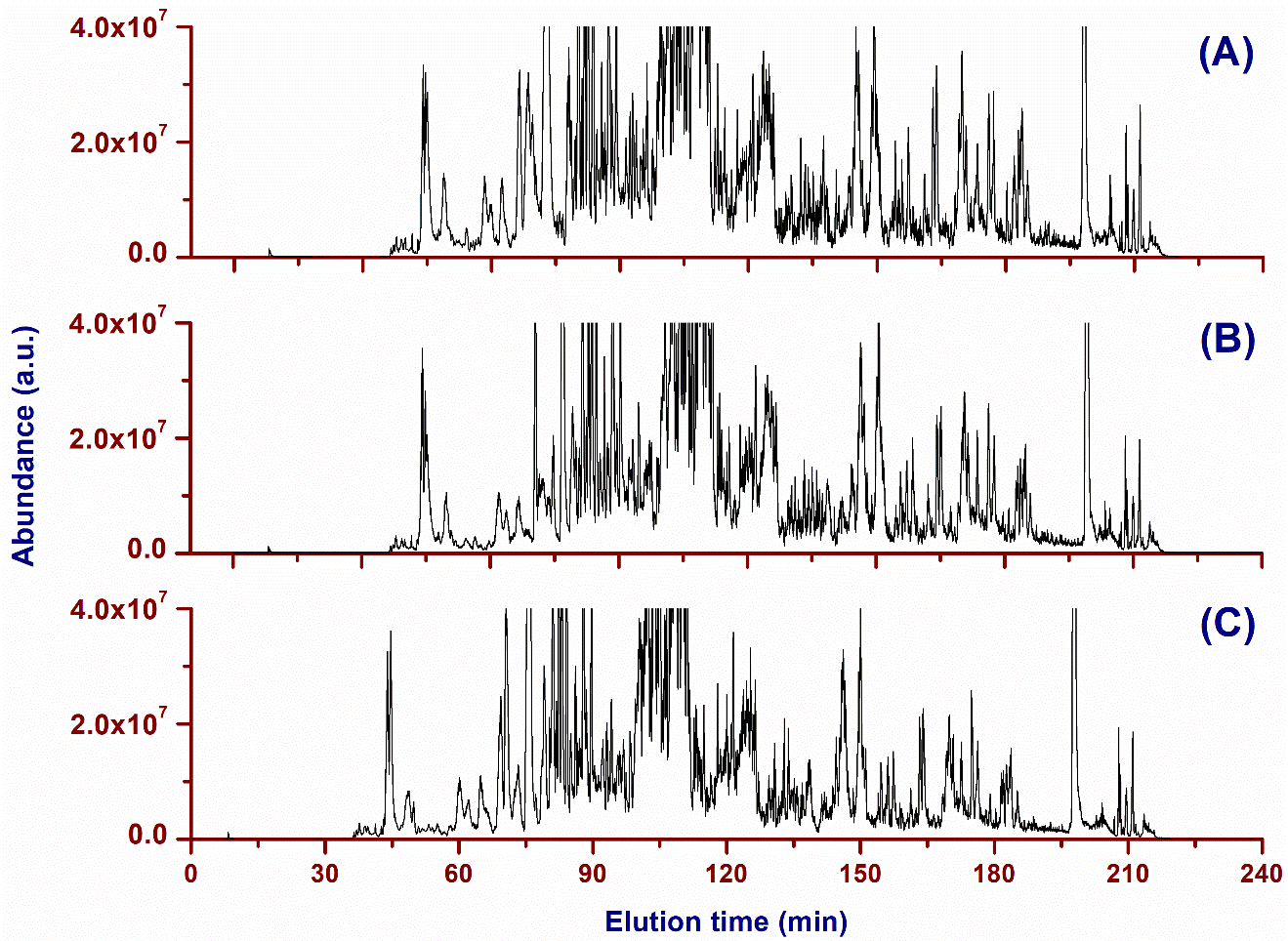


**Figure S1.** MS-only base-peak chromatograms from C18-RPLC-MS/MS analysis of the 1:1 mixture of the light- and heavy-diethylated intact N-glycopeptides from MCF-7/ADR and MCF-7/ADR CSCs. (A, B, C), three technical replicates; a.u. = arbitrary unit.


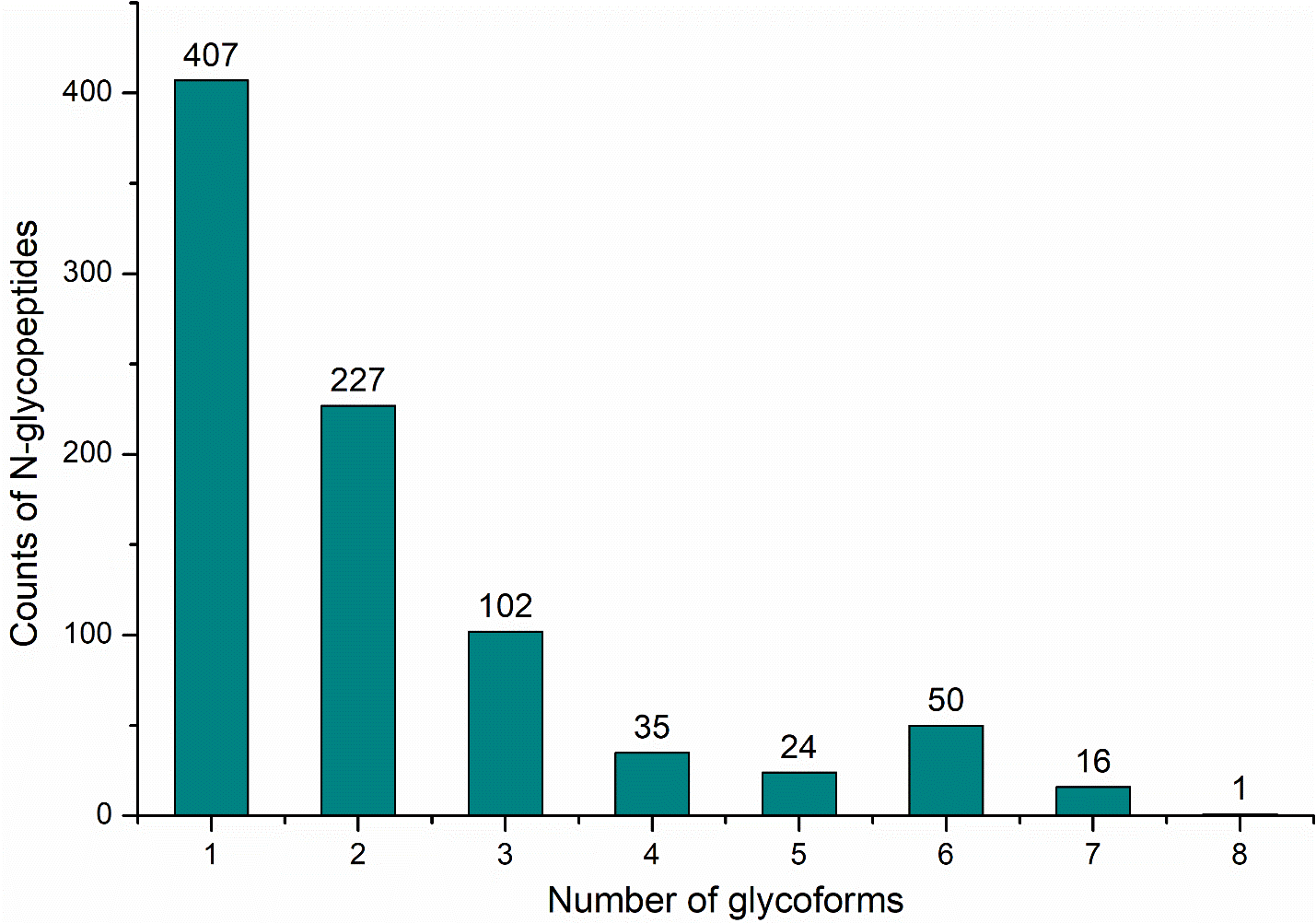


**Figure S2**. Counts of intact N-glycopeptides IDs with different number of glycoforms.


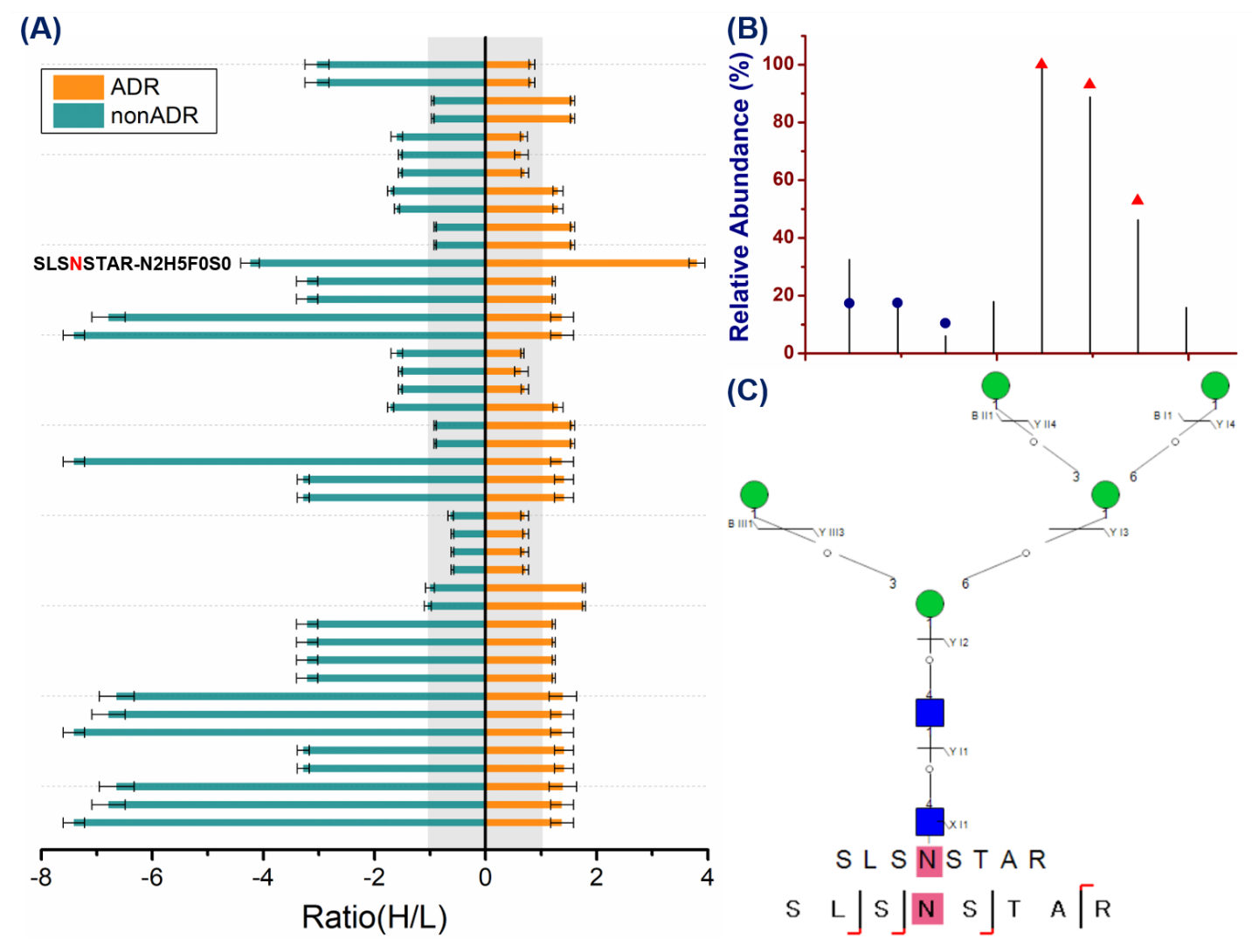


**Figure S3.** Comparison of DEGPs in MCF-7 CSCs and MCF-7/ADR CSCs. (A) Error bar of ratio(heavy/light) in these two cell lines (both observed in two out of three technical replicates). (B) the isotopic envelope fingerprinting maps of the precursor ions, (C) selective fragmentation and the graphical fragmentation map of N-glycan moiety with the peptide backbone and the peptide backbone with one core GlucNAc of intact N-glycopeptide SLSNSTAR-N2H5F0S0 (Serpin H1, P50454, N120).


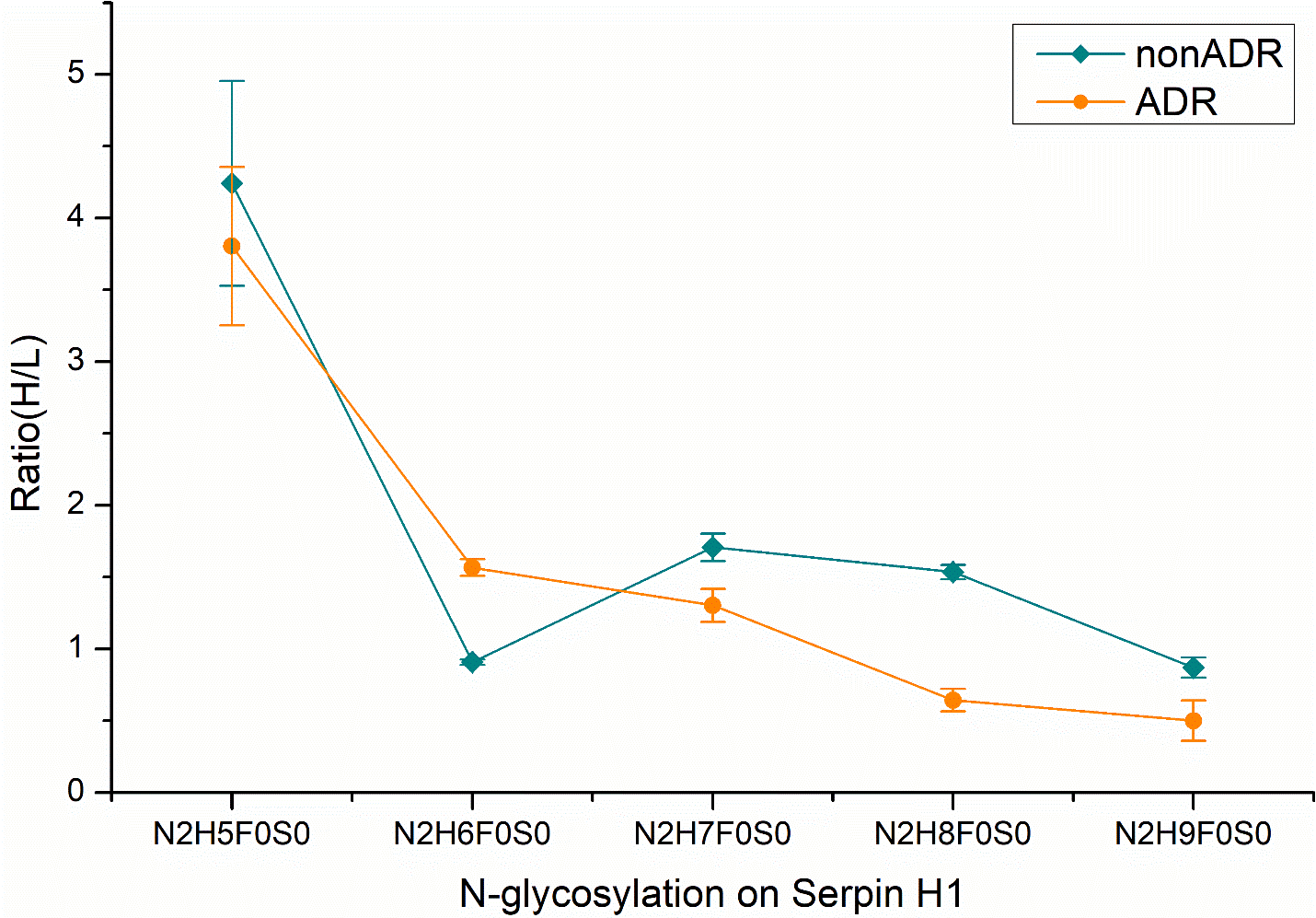


**Figure S4.** Error curve of differentially expressed ratio(heavy/light) of five high-mannose intact N-glycopeptide SLSNSTAR_N2HxF0S0 (x=5, 6, 7, 8, 9) on N-glycosite N120 of intact N-glycoprotein serpin H1 (P50454) in both MCF-7 CSCs and MCF-7/ADR CSCs.

| 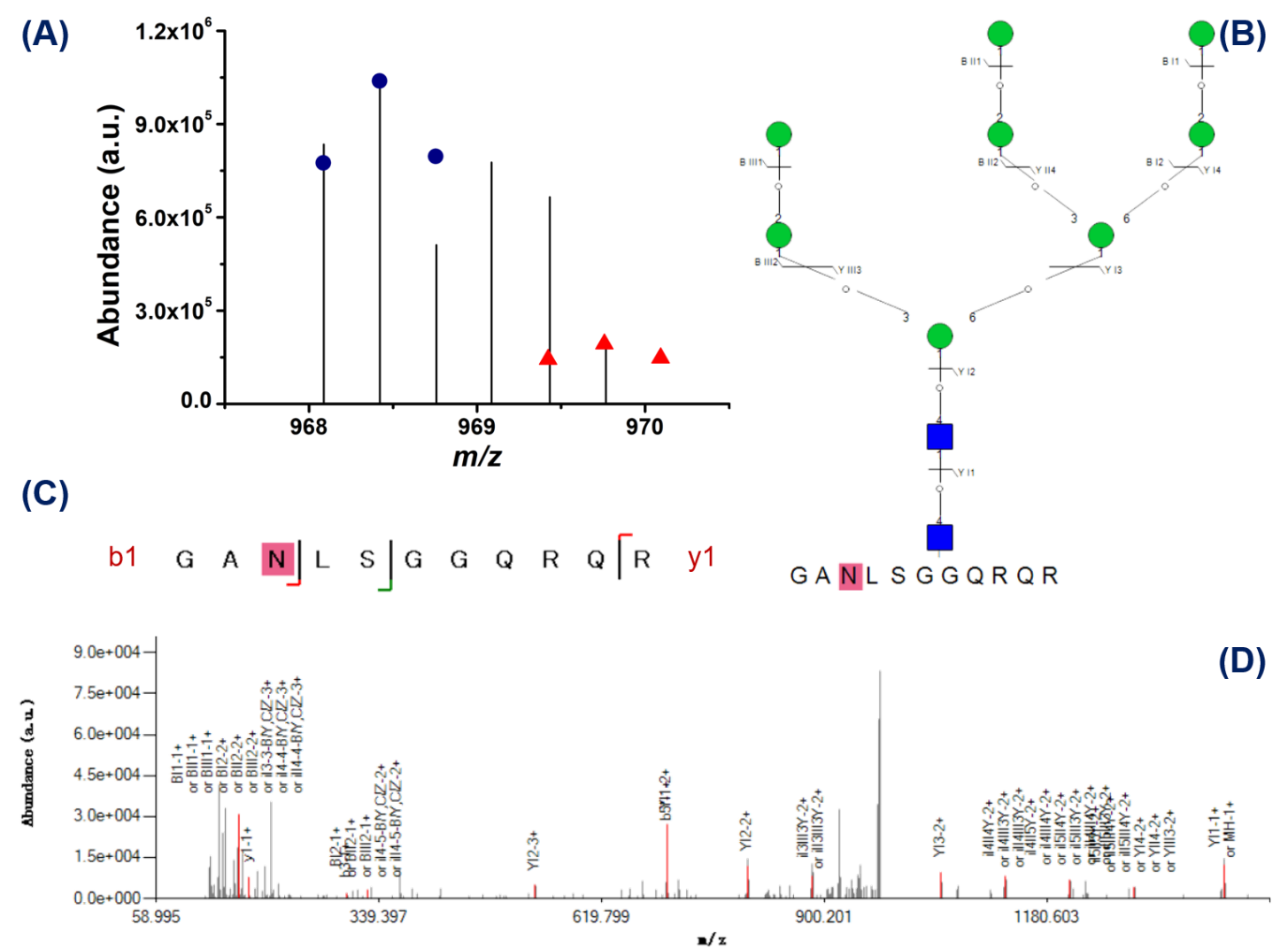 | **Figure S5.** Identification of intact N-glycopeptide GANLSGGQRQR-N2H8F0S0 from multidrug resistance-associated protein 5 (ABCC5, MRP5_HUMAN, O15440, N684) from spectrum 17391 of TR1, down-regulation of 0.46-fold in MCF-7/ADR CSCs vs. MCF-7/ADR cells was observed with the left two isotopic peaks. (A) the isotopic envelope fingerprinting maps of the precursor ions, (B) selective fragmentation and the graphical fragmentation map of N-glycan moiety with the peptide backbone, (C) fragmentation and the graphical fragmentation map of the peptide backbone with one core GlcNAc, and (D) the annotated MS/MS spectrum with the matched fragment ions. |
| --- | --- |

| 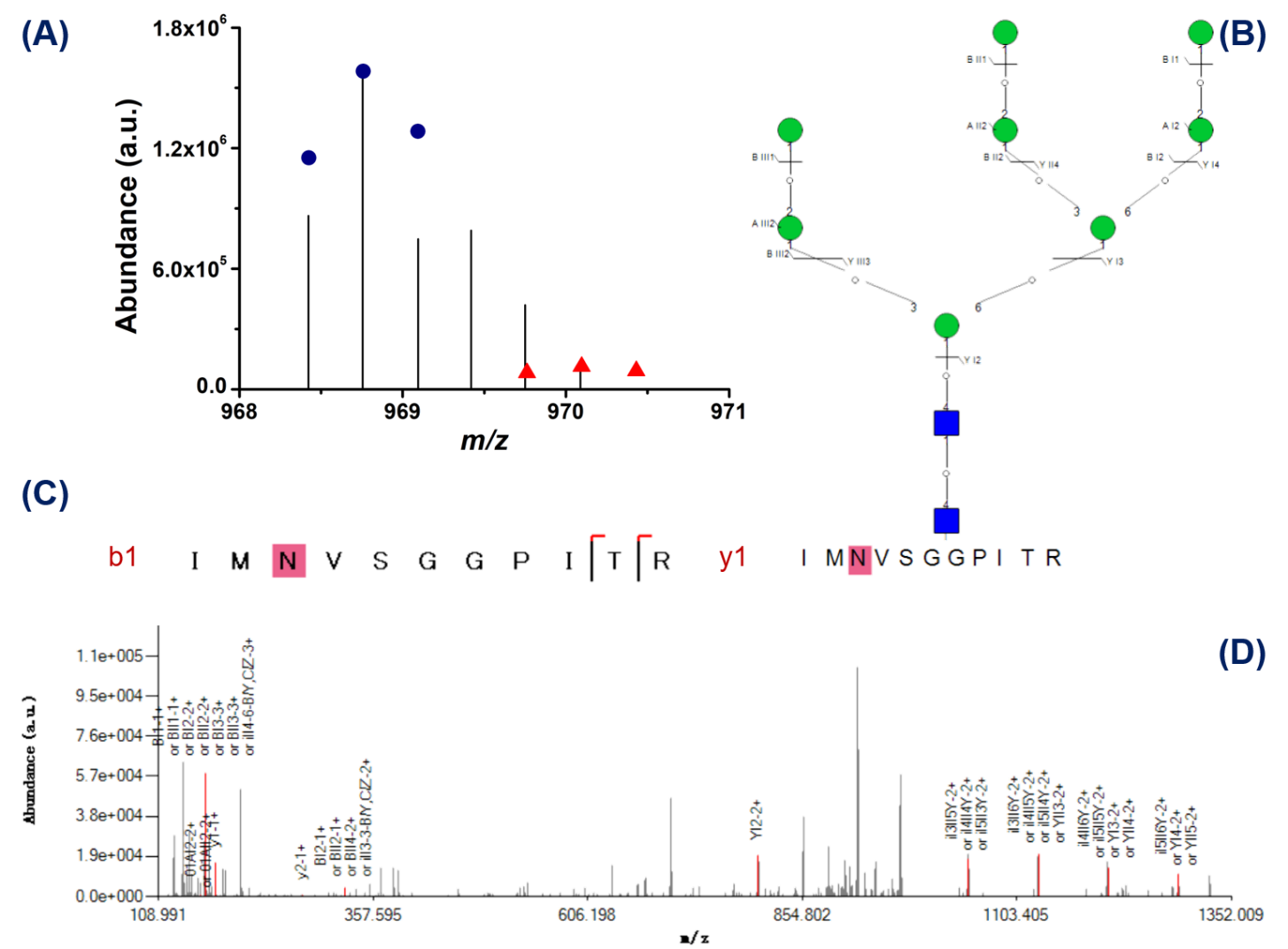 | **Figure S6.** Identification of intact N-glycopeptide IMNVSGGPITR-N2H8F0S0 from retinal-specific ATP-binding cassette transporter (ABCA4_HUMAN, P78363, N1588) from spectrum 17575 of TR2, down-regulation of 0.22-fold in MCF-7/ADR CSCs vs. MCF-7/ADR cells was observed with the left two isotopic peaks. (A) the isotopic envelope fingerprinting maps of the precursor ions, (B) selective fragmentation and the graphical fragmentation map of N-glycan moiety with the peptide backbone, (C) fragmentation and the graphical fragmentation map of the peptide backbone with one core GlcNAc, and (D) the annotated MS/MS spectrum with the matched fragment ions. |
| --- | --- |

| 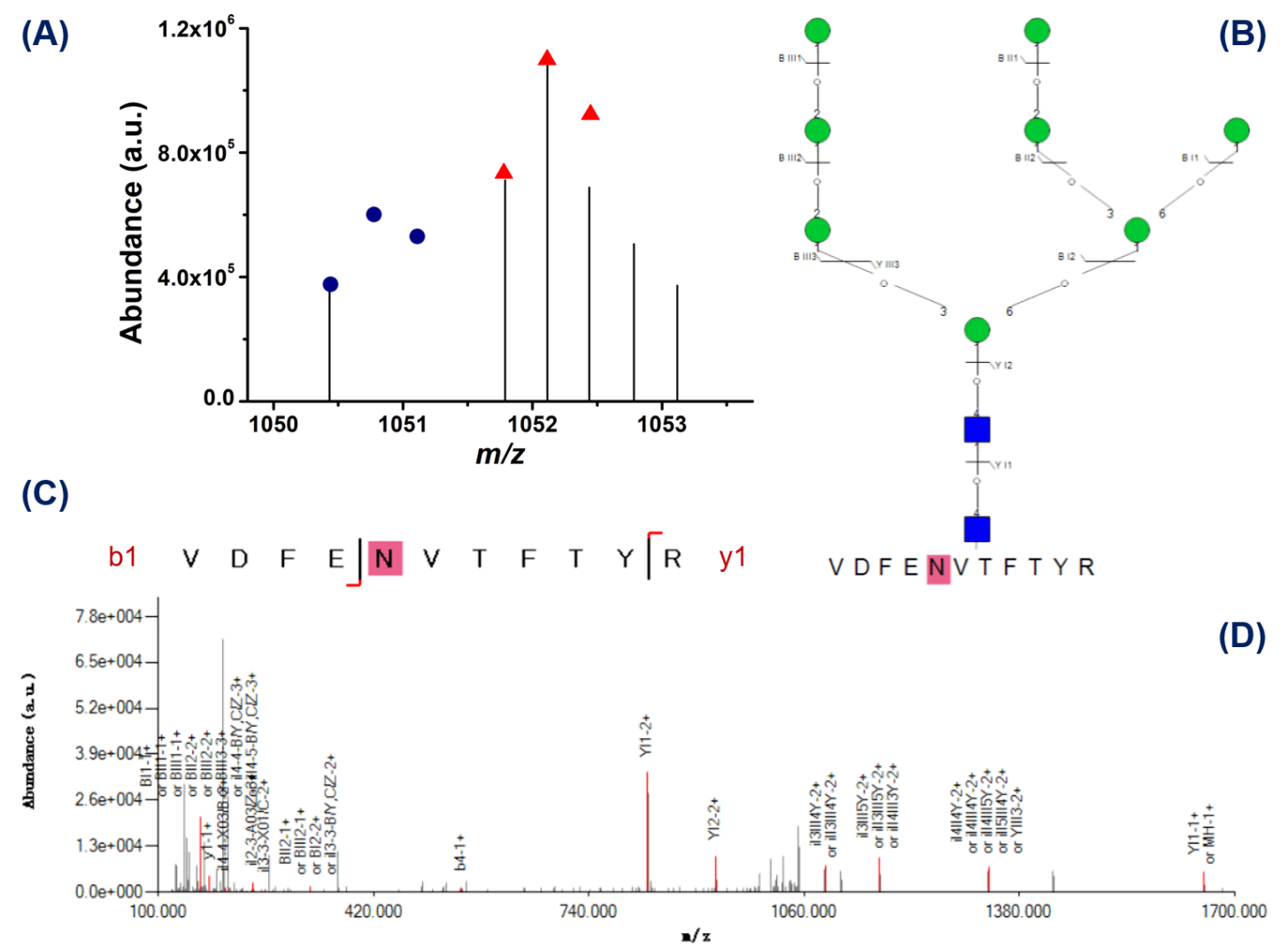 | **Figure S7.** Identification of intact N-glycopeptide VDFENVTFTYR-N2H8F0S0 from ATP-binding cassette sub-family B member 9 (ABCB9_HUMAN, Q9NP78, N508) from spectrum 20416 of TR3, up-regulation of 1.89-fold was observed in MCF-7/ADR CSCs vs. MCF-7/ADR cells as quantitated with the left isotopic peak. (A) the isotopic envelope fingerprinting maps of the precursor ions, (B) selective fragmentation and the graphical fragmentation map of N-glycan moiety with the peptide backbone, (C) fragmentation and the graphical fragmentation map of the peptide backbone with one core GlcNAc, and (D) the annotated MS/MS spectrum with the matched fragment ions. |
| --- | --- |
|  |  |


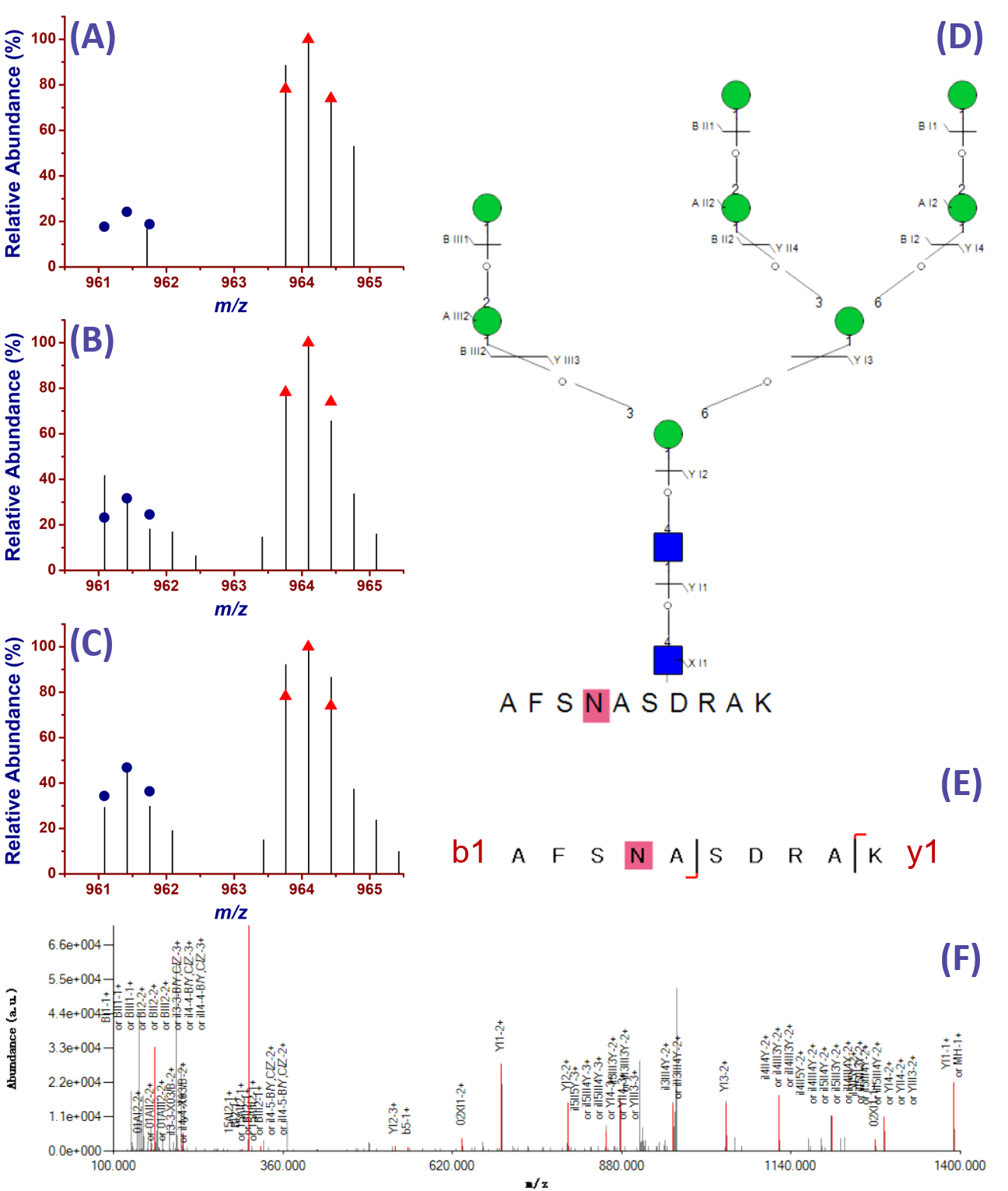


**Figure S8.** Quantification of up-regulation (2.66±0.03) of intact N-glycopeptide AFSNASDRAK-N2H8F0S0 (Zinc finger protein GLI1, P08151, N-glycosite N344) in MCF-7/ADR CSCs relative to MCF-7/ADR. (A, B, C) the isotopic envelope fingerprinting maps of the precursor ions in the three technical replicates; (D) selective fragmentation and the graphical fragmentation map of N-glycan moiety with the peptide backbone, (E) fragmentation and the graphical fragmentation map of the peptide backbone with one core GlcNAc, and (F) the annotated MS/MS spectrum with the matched fragment ions in representative spectrum 17226 of TR1.


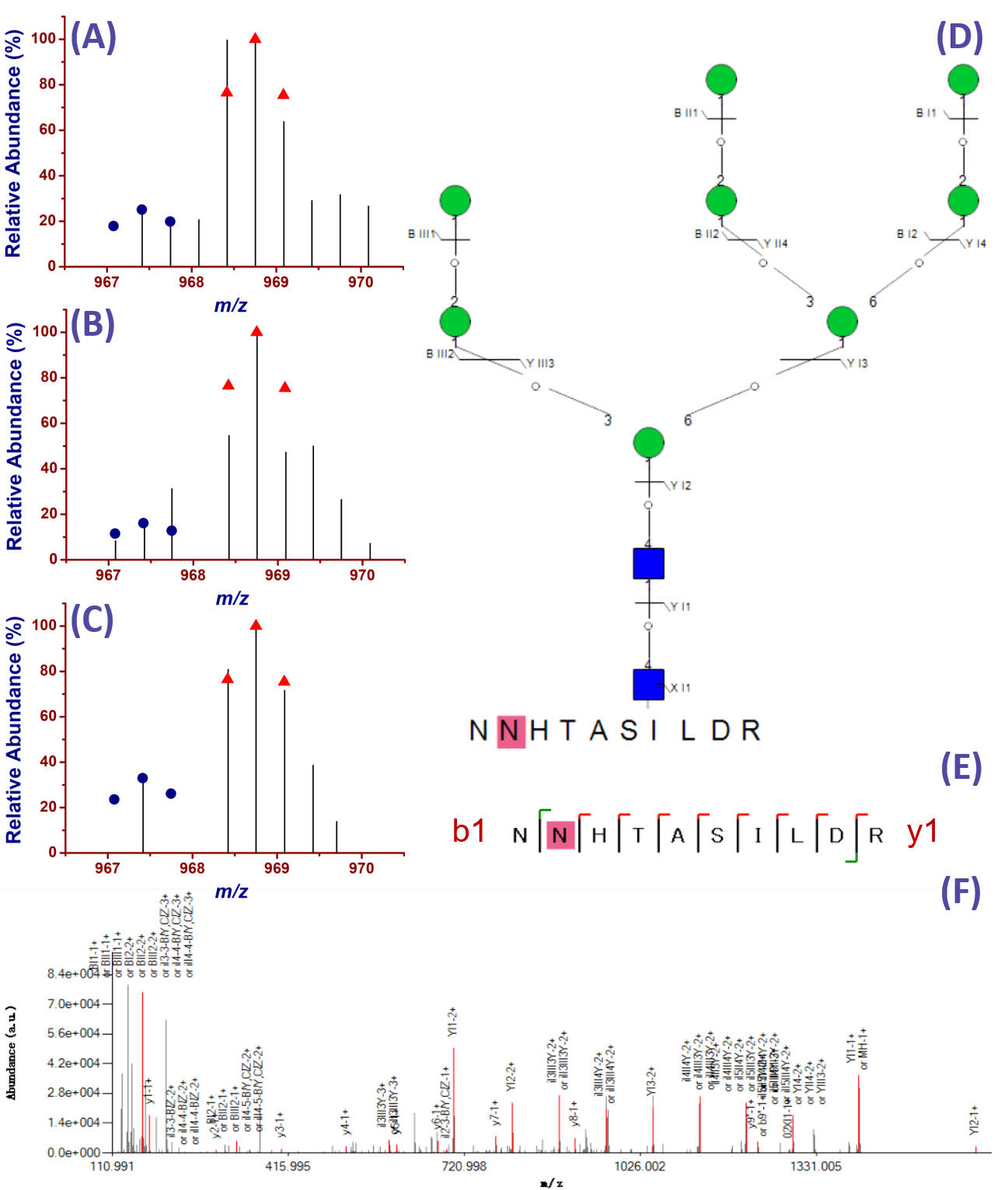


**Figure S9.** Quantification of up-regulation (3.39±0.26) of intact N-glycopeptide NNHTASILDR-N2H8F0S0 (CD63 antigen, P08962, N-glycosite N130) in MCF-7/ADR CSCs relative to MCF-7/ADR. (A, B, C) the isotopic envelope fingerprinting maps of the precursor ions in the three technical replicates; (D) selective fragmentation and the graphical fragmentation map of N-glycan moiety with the peptide backbone, (E) fragmentation and the graphical fragmentation map of the peptide backbone with one core GlcNAc, and (F) the annotated MS/MS spectrum with the matched fragment ions in representative spectrum 17200 of TR1.


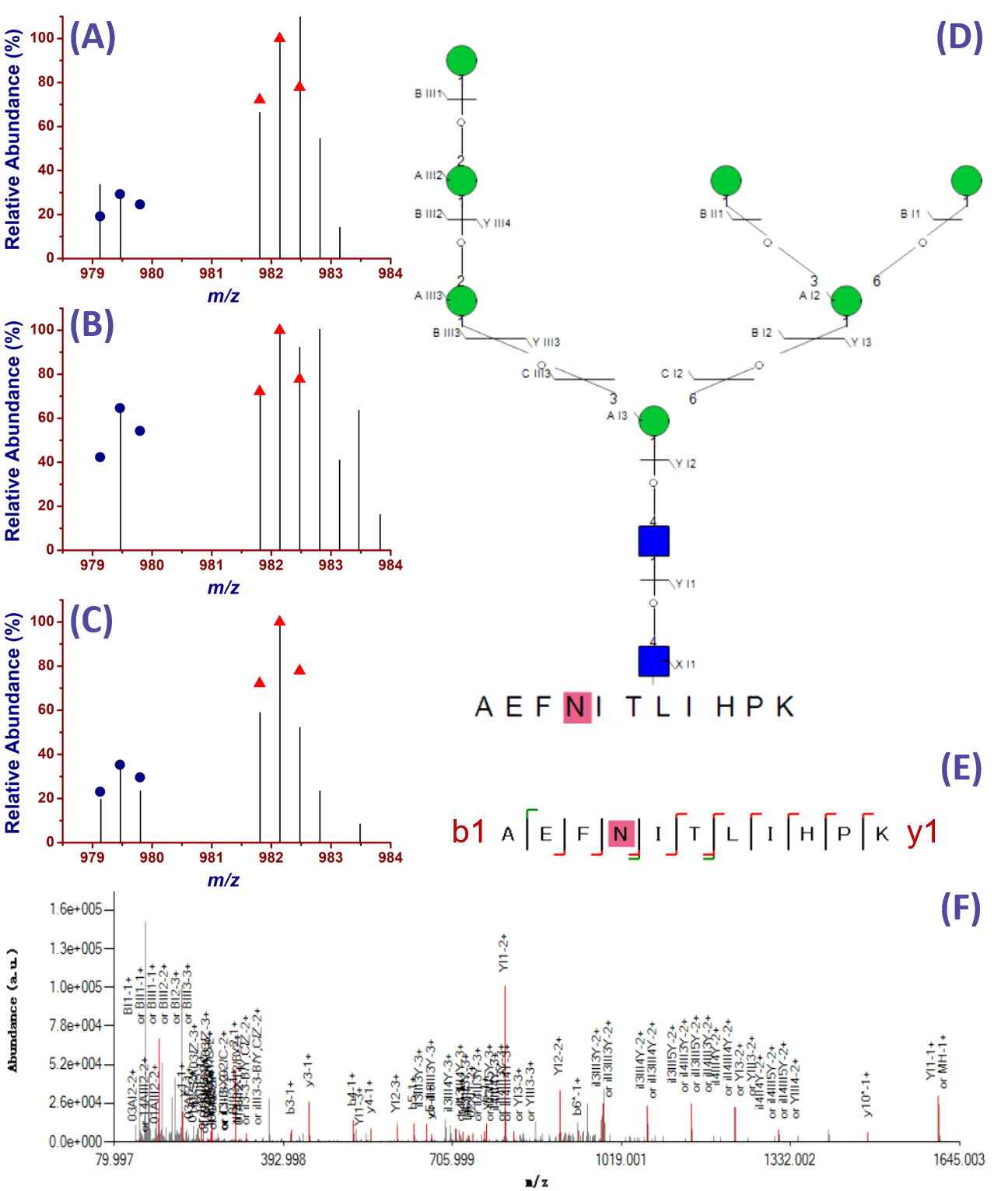


**Figure S10.** Quantification of up-regulation (2.30±0.53) of intact N-glycopeptide AEFNITLIHPK-N2H7F0S0 (CD13, P15144, N-glycosite N234) in MCF-7/ADR CSCs relative to MCF-7/ADR. (A, B, C) the isotopic envelope fingerprinting maps of the precursor ions in the three technical replicates; (D) selective fragmentation and the graphical fragmentation map of N-glycan moiety with the peptide backbone, (E) fragmentation and the graphical fragmentation map of the peptide backbone with one core GlcNAc, and (F) the annotated MS/MS spectrum with the matched fragment ions in representative spectrum 26814 of TR1.


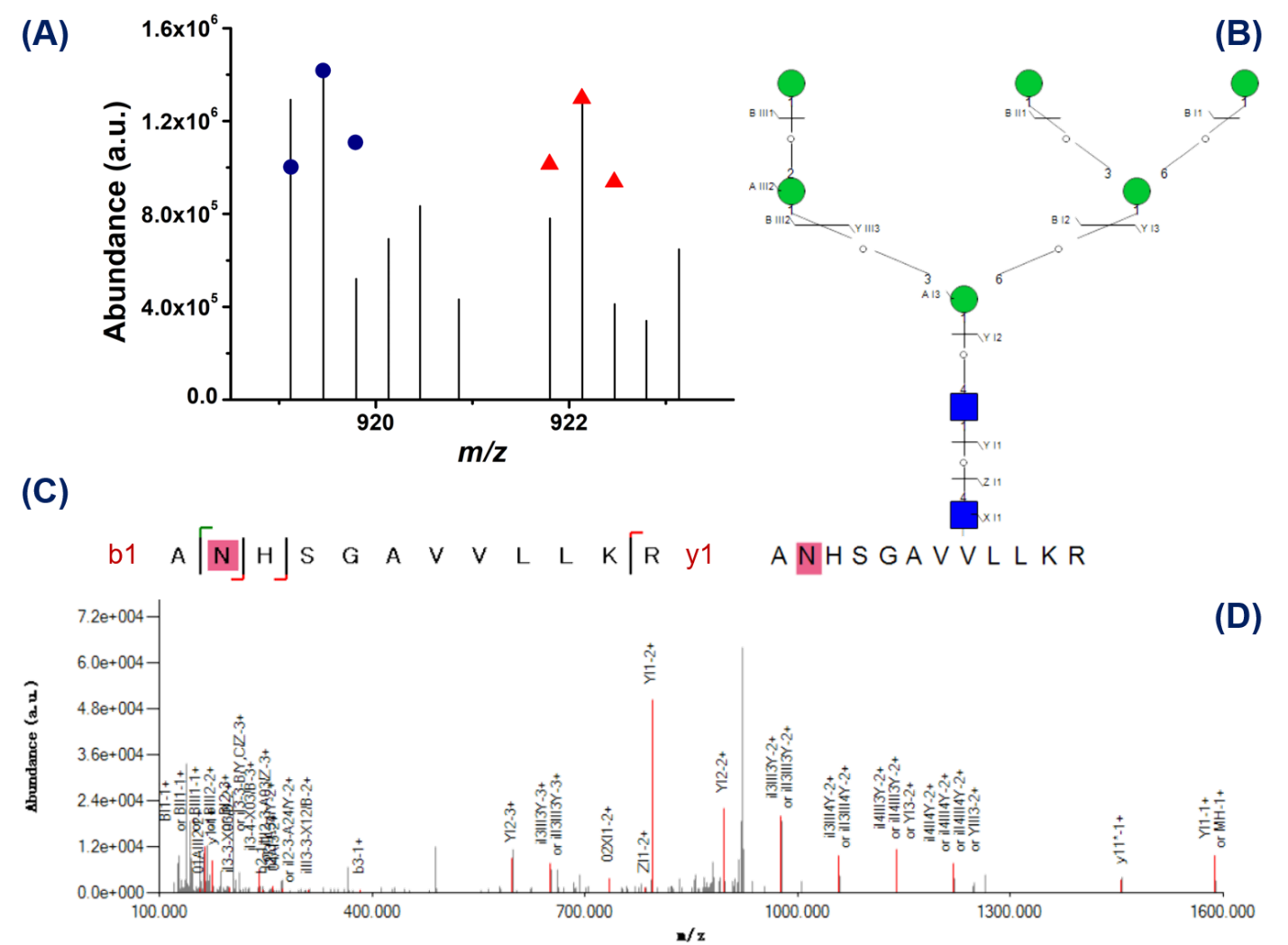


**Figure S11.** Identification of intact N-glycopeptide ANHSGAVVLLKR-N2H6F0S0 from Integrin alpha-6 (CD49F, P23229, N323) from spectrum 19068 of TR2, down-regulation of 0.77-fold was observed in MCF-7/ADR CSCs vs. MCF-7/ADR cells. (A) the isotopic envelope fingerprinting maps of the precursor ions, (B) selective fragmentation and the graphical fragmentation map of N-glycan moiety with the peptide backbone, (C) fragmentation and the graphical fragmentation map of the peptide backbone with one core GlcNAc, and (D) the annotated MS/MS spectrum with the matched fragment ions.


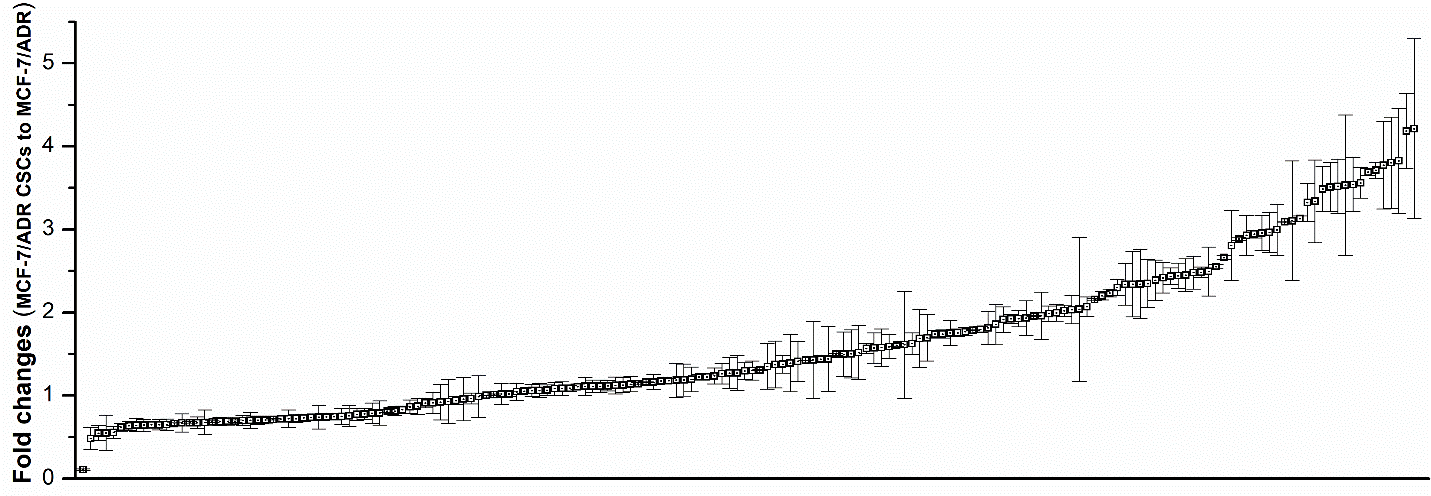


**Figure S12**. Box plot of fold changes in glycopeptides from MCF-7/ADR CSCs relative to MCF-7/ADR.
